# Evaluating the Effectiveness of Wildlife Detection and Observation Technologies at a Solar Power Tower Facility

**DOI:** 10.1371/journal.pone.0158115

**Published:** 2016-07-27

**Authors:** Robert H. Diehl, Ernest W. Valdez, Todd M. Preston, Michael J. Wellik, Paul M. Cryan

**Affiliations:** 1 U.S. Geological Survey (USGS), Northern Rocky Mountain Science Center, Bozeman, Montana, United States of America; 2 USGS, Fort Collins Science Center, Fort Collins, Colorado, United States of America; 3 USGS, Upper Midwest Environmental Science Center, La Crosse, Wisconsin, United States of America; University of South Carolina, UNITED STATES

## Abstract

Solar power towers produce electrical energy from sunlight at an industrial scale. Little is known about the effects of this technology on flying animals and few methods exist for automatically detecting or observing wildlife at solar towers and other tall anthropogenic structures. Smoking objects are sometimes observed co-occurring with reflected, concentrated light (“solar flux”) in the airspace around solar towers, but the identity and origins of such objects can be difficult to determine. In this observational pilot study at the world’s largest solar tower facility, we assessed the efficacy of using radar, surveillance video, and insect trapping to detect and observe animals flying near the towers. During site visits in May and September 2014, we monitored the airspace surrounding towers and observed insects, birds, and bats under a variety of environmental and operational conditions. We detected and broadly differentiated animals or objects moving through the airspace generally using radar and near solar towers using several video imaging methods. Video revealed what appeared to be mostly small insects burning in the solar flux. Also, we occasionally detected birds flying in the solar flux but could not accurately identify birds to species or the types of insects and small objects composing the vast majority of smoking targets. Insect trapping on the ground was somewhat effective at sampling smaller insects around the tower, and presence and abundance of insects in the traps generally trended with radar and video observations. Traps did not tend to sample the larger insects we sometimes observed flying in the solar flux or found dead on the ground beneath the towers. Some of the methods we tested (e.g., video surveillance) could be further assessed and potentially used to automatically detect and observe flying animals in the vicinity of solar towers to advance understanding about their effects on wildlife.

## Introduction

**Fig 1:**
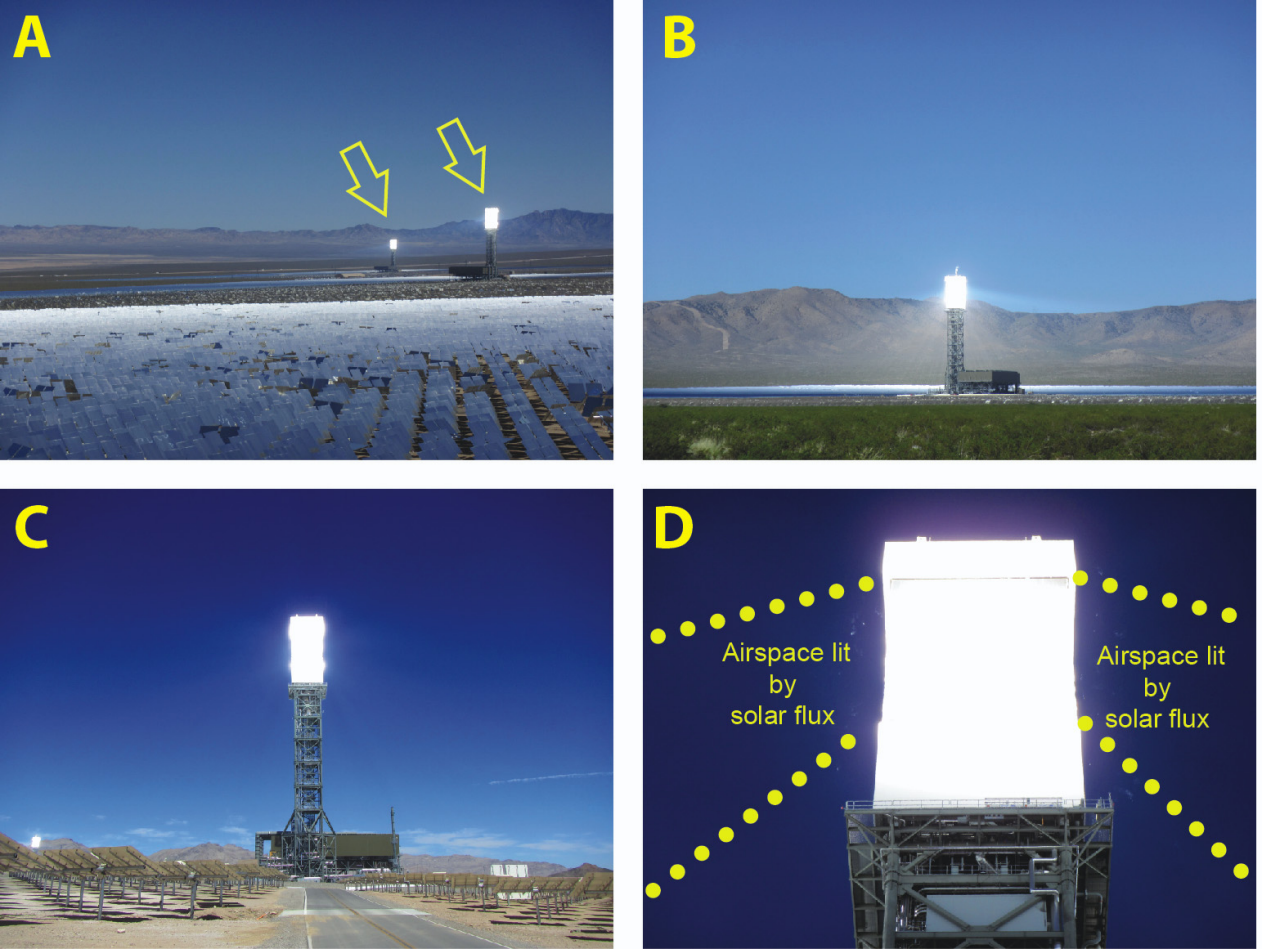
Different views of a solar power tower facility. Images of the solar power towers: A) Far view of a solar tower facility showing a foreground of mirror “heliostats” used to reflect sunlight toward the “receiver” towers (yellow arrows). B & C) Closer views (approximately 2 km and 400m away, respectively) of towers being lit with sunlight, as well as the machinery associated with power generation. D) Top of a solar power tower being heated by solar flux and multiple, small smoking objects in the closely surrounding airspace. For scale, the illuminated receiver (white rectangle) measures approximately 40 m in height and 15 m in width. Photos by Paul Cryan, USGS.

Solar thermal energy facilities incorporating mirrors and concentrating towers (hereafter ‘solar towers’) produce electrical energy from sunlight at an industrial scale. These facilities generate electricity using an array of large, pivoting mirrors, called heliostats, to concentrate energy from the sun onto a “receiver” at the top of a central heat-absorbing tower (Fig 1). The receiver heats water inside the tower, creating steam that is then piped to a conventional turbine to generate electricity. Sunlight reflected from heliostats toward the tower is referred to as “solar flux.” Solar flux is not thermal energy but rather concentrated light, and the intensity of flux is greatest at the tower receiver, where surface temperatures can reach more than 500°C. As radiant energy, flux does not substantially heat air through which it travels. Rather, solar flux is converted to heat energy when striking an object, which can be problematic for animals flying through sufficiently powerful flux fields.

Solar towers represent emerging technology with the potential to become a substantial source of renewable energy, but because this technology is new there is uncertainty about its potential effects on flying animals. During 1982–83, McCrary et al. [1] studied bird mortality at a 10-megawatt (MW) solar tower facility with a single 86-m-tall tower in the Mojave Desert of southern California, USA. During about 40 weeks of monitoring, 70 bird fatalities representing 26 species were found [1]. The majority (80%, n = 57) of fatalities likely resulted from collisions with structures (75% with heliostats and 25% with the tower), and 19% (n = 13) of the birds apparently died from burn injuries [1]. Although direct observations of birds being burned were not made during that study, the researchers assumed that burn injuries were sustained in the solar flux around the receiver and at so-called “standby areas”—locations in the airspace where mirrors concentrate sunlight when not focused on the tower [1]. All seven species of birds found with burn injuries were migratory species that fed on insects to some degree. Insect presence in the solar flux was not noted, but McCrary et al. [1] speculated that aerial-foraging insectivorous birds (e.g., swifts and swallows) were “apparently more susceptible to this form of mortality because of their feeding behavior”.

Mortality of birds attributable to collisions and burn injuries was later documented when the world’s largest solar tower facility to date became operational in the Mojave Desert during 2014 [2,3]. Although this newer technology does not require standby areas distant from the tower, as at the site studied by McCrary et al. (Ivanpah Solar Electric Generating System, personal communication), smoking objects, including insects and birds, were also observed in the solar flux-illuminated airspace closely surrounding these new solar towers. The identity and source of some of these objects at new solar towers has been difficult to visually determine from the ground. The lack of previous studies at solar towers limits our ability to interpret such observations or anticipate the extent to which this unique technology might affect flying animals.

Several solar tower facilities are either built, under construction, or in the planning stages in the North American Desert Southwest, as well as elsewhere in the world (www.wipp.energy.gov/science/energy/powertower.htm; accessed 19 February 2015). The large size of some of these newer facilities (e.g., >40 times the land area and about 1.5 times the tower height of the site studied by [1]) and brightness of the receivers under solar flux conditions present challenges to traditional monitoring methods, such as ground-based observers, and encourage investigation into additional ways of effectively detecting and observing animals flying near solar towers and in surrounding airspace. Detecting, observing, identifying, and documenting small animals flying high above the ground around tall structures can be challenging, yet sometimes advanced monitoring technologies can help achieve these goals (e.g., [4,5]).

In this observational pilot study, we worked at the world’s largest existing solar tower facility to assess the efficacy of using radar, videography, and insect sampling to detect and observe the daytime and nighttime presence, diversity, movement and behavior of birds, bats, and insects near operating solar towers. We did not take a quantitative approach or test hypotheses due to incomplete understanding of these phenomena, lack of existing field-tested research methods, and available time to conduct this pilot study. Rather, we experimented with potential tools and techniques for detecting and observing aerial fauna that could be used in future research or monitoring efforts. Over the course of two week-long observation periods in spring and autumn of 2014, our specific objectives were to evaluate the performance of these methods and determine which would be appropriate for future use to quantify the presence, diversity, movement, and behavior of animals flying near solar towers.

## Materials and Methods

We conducted this pilot study at the Ivanpah Solar Electric Generating System (ISEGS) in southeastern California, USA. This work was conducted under USGS Technical Assistance Agreement #14SWRTAARB003561 with ISEGS providing permission for access to its facilities during the study. This study did not involve endangered or protected species, and it did not include handling, manipulating, or influencing the behaviors of birds, bats, or any other vertebrates. Live insects were captured and killed as part of the study, however insects are not covered under the Animal Welfare Act and thus not subject to IACUC review.

ISEGS is located in the Ivanpah Valley of San Bernardino County near the border of California and Nevada (35° 33’N, 115° 28’W), approximately 75 km SSE of Las Vegas and 300 km NE of Los Angeles. The surrounding biotic community is Mojave Desert Scrub [6], with the exception of a large playa lake and a golf course that includes several ponds, both within 10 km of the facility to the east. The facility, which has a combined installed energy generating capacity of 377 MW, covers just over 1,400 ha of public land, and includes approximately 170,000 computer-controlled heliostats that are used to direct sunlight toward three central towers (www.ivanpahsolar.com; accessed 19 February 2015). Each tower stands 140 m above ground level (AGL), is illuminated at night by obstruction and security lighting, is approximately 15 m by 15 m in cross section and is self-supporting, and has a receiver section (where heliostats aim solar flux) spanning from approximately 100–136 m AGL. Solar flux around each tower generally occurs in a hollow-cone-shaped area extending from the bottom of the receiver toward the ground at an angle of approximately 30° from vertical, and from the top of the receiver toward the ground at approximately 70° (Fig 1D). Within this region during power generation, solar flux increases in intensity as distance to the receiver decreases.

**Fig 2:**
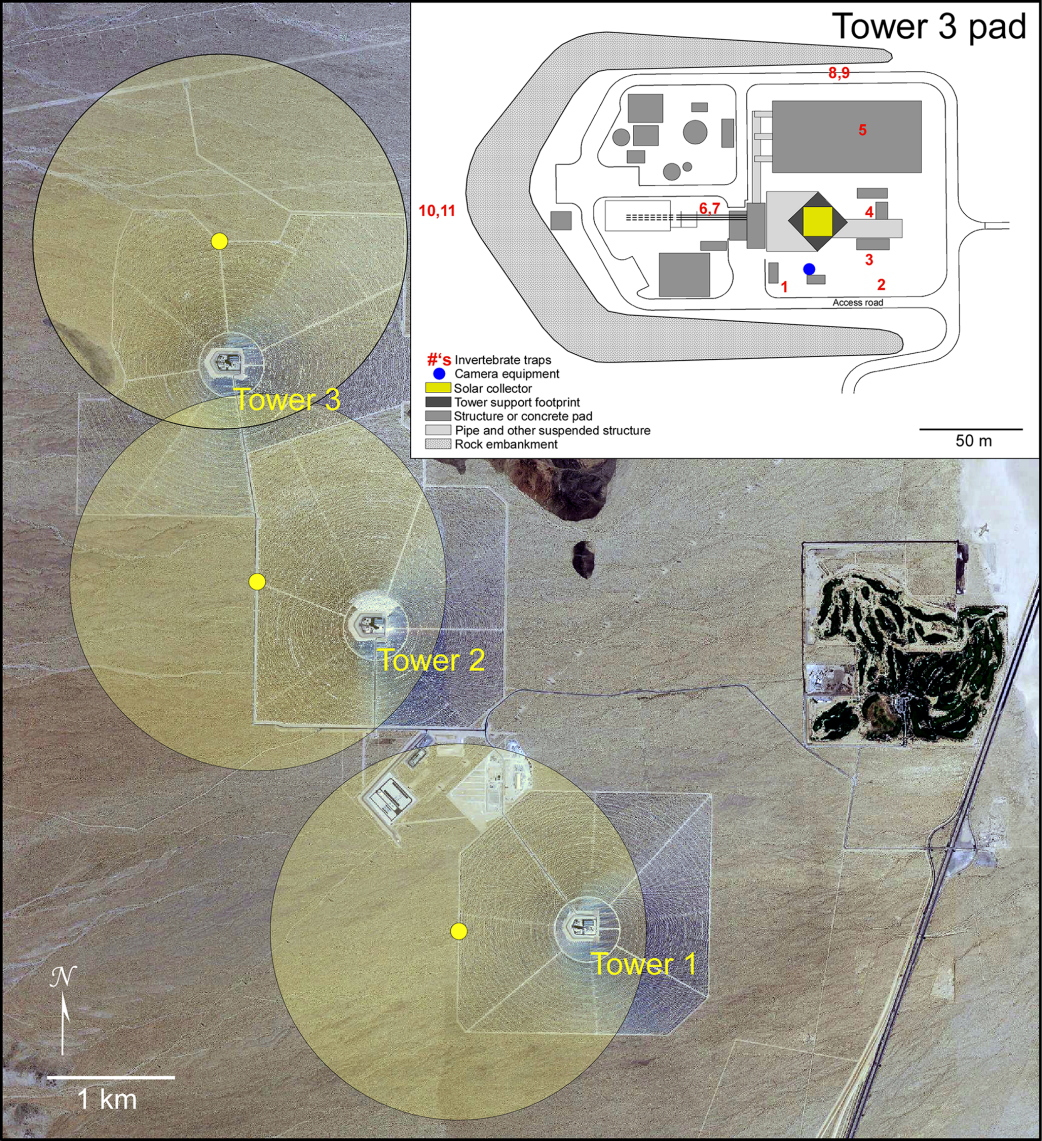
Aerial view of the solar facility where study was conducted. Location of each solar concentrating tower is indicated in the aerial view of facility, along with radar locations and coverage (yellow dots and shaded circles). The inset shows an expanded view of the Tower 3 pad (all towers share same layout), with structures in shades of gray as well as the locations of the concentrating tower (yellow), insect traps (red), and camera location (blue).

We conducted our study at ISEGS during site visits from 14–22 May and 2–11 September 2014, representing spring and autumn periods of bird, insect, and bat migration and seasonal abundance. During the May visit all monitoring occurred at Tower 3. During 2–4 September we began our monitoring at Tower 2, but after 4 September video cameras and radar equipment were moved to Tower 1 because Tower 2 was taken offline for repair. Video and radar monitoring continued at Tower 1 from 5–11 September, whereas insect trapping continued at Tower 2 through the end of the September visit, even though the tower was operating intermittently (limited solar flux) after 3 September. Fig 2 shows the locations of the radar units relative to the towers and the arrangement of video cameras and insect traps at each tower base. Although tower locations changed, the locations of video cameras and insect traps were identical in relation to the focal tower.

## Radar monitoring

We used a modified 25-kW x-band radar (Model Furuno FR2127, Furuno USA, Inc., Camas, Washington) refitted with a parabolic 2.1° half-power beam-width antenna to gather data on the presence and diversity of aerial fauna in the ISEGS airspace [7]. The portable radar system was deployed at three locations primarily in response to operational conditions at the facility and ran nearly continuously. At each location we positioned the radar to limit ground clutter while maximizing visibility of the solar towers. During 14–22 May, the radar was positioned north of Tower 3; during 3–7 September the radar was west of Tower 2; and during 7–11 September the radar was west of Tower 1. Fig 2 shows these radar locations and associated coverage areas out to 1,500 m range. Antenna elevation was adjusted to accommodate variations among locations in terrain and distance from the tower. Antenna elevations were 6.5°, 4.2°, and 9.0° at Towers 1, 2, and 3, respectively. During normal operation, the radar rotates through 360° every 2.5 sec at which point the locations of radar targets are digitally captured and stored as raster bitmap imagery.

Target track profiles were developed with the program radR [8], using a modified video plugin. To do this, bitmap images were first converted to portable network graphics (png) files. A subset of these files bracketing 20-min periods centered on the top of each hour were then merged using the program ffmpeg (v0.6.7) into grayscale MP4 videos. Our modified video plugin extracted data on frame size and rate, which were then used in radR’s target identification and track development algorithms. Parameter settings used by radR for target identification and track development were established by comparing output against original bitmap imagery. We only accepted tracks involving four or more sequential target detections in order to increase the likelihood of obtaining reliable track metrics and reduce the likelihood of spurious observations, usually caused by radar clutter. Using output from radR, track summaries for each target included start time, duration, length, ground speed, direction, and altitude [8].

**Fig 3:**
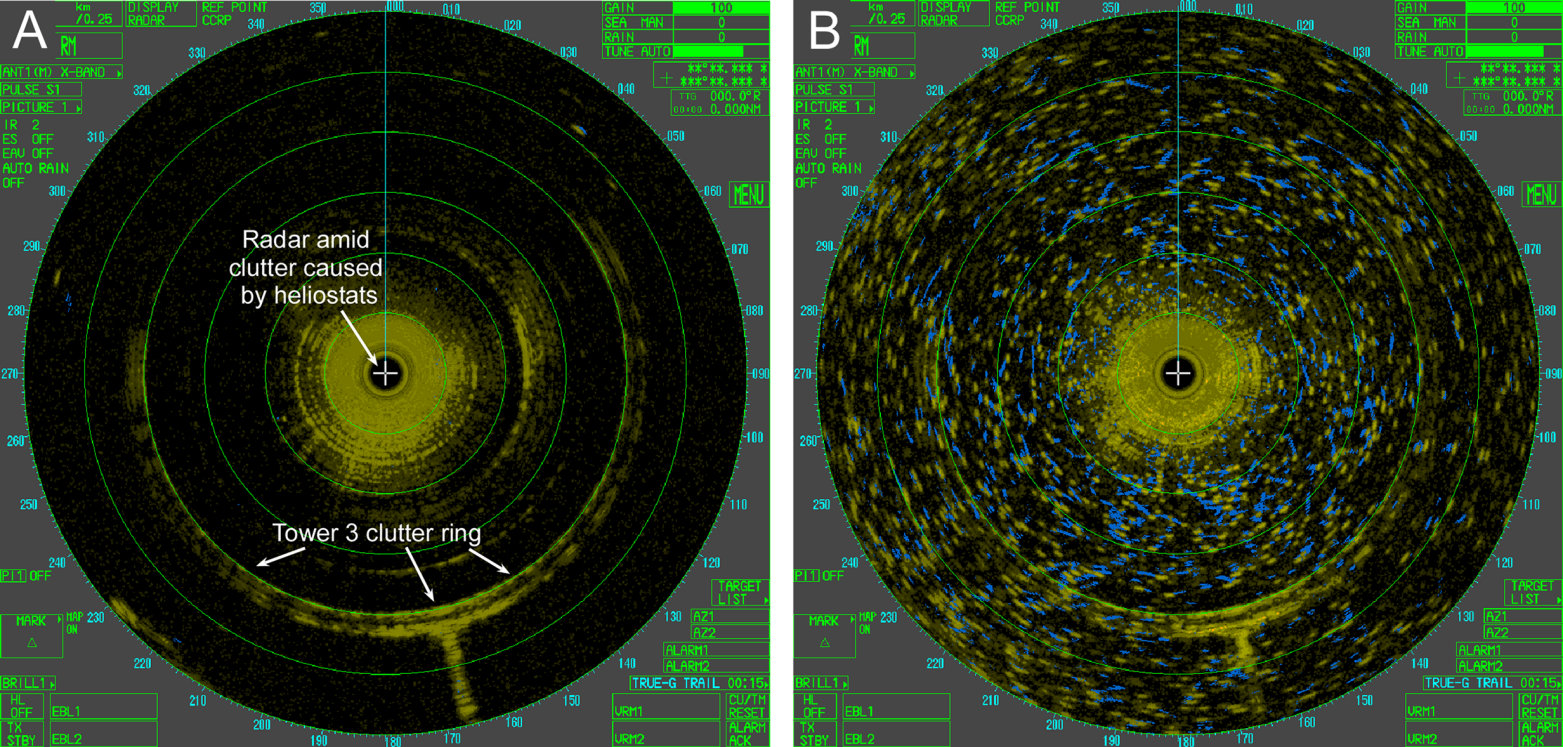
Examples of radar-monitored airspace around a solar tower. Sweeps of the radar at 0600 PDT (A) and 1100 PDT (B) on 16 May 2014 while operating near Tower 3. Range rings in green denote 250-m intervals out to 1,500 m. The radar is located in the center of the image and is surrounded by ground clutter caused primarily by the heliostat field. Tower 3 causes discernible clutter to the SSW and throughout a ~200° ring at about 1,000 m range. Flying animals, shown as yellow dots (most recent location) with blue tracks (previous locations), were nearly absent in the airspace at 06:00 (A) but occurred in large numbers by 11:00 (B), consistent with patterns observed daily (see text). An animation of (B) is available in S1 Video.

Quality control was applied to radar data, primarily to retain biological echoes while reducing clutter. Tracks of animals flying between 500 m and 1,500 m range from the radar were retained at all three tower monitoring locations. The minimum range distance was determined by the presence of dynamic ground clutter, primarily from the heliostat field (Fig 3), within 500 m of the radar. During operation at Tower 2, considerable ground clutter occurred to the west of the radar, attributable to relief in terrain and human infrastructure. This region was also removed from analysis by excluding data between 177° and 360° within each radar sweep. Vehicle traffic within radar coverage was extremely limited and therefore rarely if ever a source of clutter. Remaining stationary clutter was further reduced by removing non-moving “tracks” generated by radR tracking algorithms. Some extremely slow moving animals may have been rejected by this process, but non-moving or extremely slow moving targets comprised a small portion of total tracks (≤0.013% of tracks showed <5 m net displacement). This helped ensure further analyses included relatively few artifacts but at the loss of some biological data. These losses probably occur with some bias, since such filters are unlikely to operate equally on different kinds of animals. Finally, small invertebrates are usually present in the airspace, but as weak Rayleigh scatterers they are often either undetectable individually [9] or, if occurring in large numbers, may be detected as a weak and un-trackable din (R. Diehl, personal observation).

We attempted to discriminate vertebrate-like targets from invertebrate-like targets by airspeed, or the speed of targets relative to the air around them, which can be computed by vector-subtracting wind velocity from ground velocity [10]. (Vertebrates are likely overwhelmingly dominated by birds and invertebrates by insects, so hereafter vertebrate-like targets are referred to as birds and invertebrate-like targets as insects.) We examined the suitability of winds from two sources. Data on wind speed and direction at the facility were provided by ISEGS staff. Within the facility, two wind stations on the ground and two 99 m up Tower 1 measure wind speed and direction every minute. We also examined surface and 900 mb (~130 m AGL) winds generated by North American Regional Reanalysis (NARR) models. Data from the pair of ISEGS surface wind stations near Tower 1 were highly correlated and proved the most consistent and plausible. Data from the pair of wind stations on the Tower 1 structure were not correlated and exhibited systematic bias. NARR wind data were not correlated with the local wind data, and being regional, were not considered further. Radar targets with airspeeds ≥7 m∙s^-1^ were considered birds, whereas targets with airspeeds <7 m∙s^-1^ were considered insects [11]. Such discrimination is inexact; some insects, including dragonflies which were frequently observed at the facility during September, show powered fight sufficient to exceed the 7 m∙s^-1^ airspeed threshold for insects.

These processing steps generate a largely clutter-free enumeration of the number of tracks, categorized by taxa (bird or insect), for each 20-min period. Results are descriptive and references to the number of tracks or abundances are considered indices of activity in the airspace rather than absolute measures.

### Video monitoring

We experimented with detecting and observing animals flying within 10–30 m of the towers using cameras capable of imaging in both very bright and low-light conditions (S1 Fig). We used five types of cameras to record digital imagery of solar towers during our visits. The first of these was a weatherproof surveillance video camera (Model Q1921-E with 19-mm and 35-mm lens, Axis Communications, Lund, Sweden) that imaged long-wavelength infrared light (9,000–14,000 nm). This thermal surveillance (TS) camera was operated continuously day and night, usually with the 35-mm lens, and imaged independently of variable illumination conditions. The second type of camera was a scientific-grade thermal (SGT) camera (Model SC8343HD, FLIR Systems, Billerica, Massachusetts, USA) that was equipped with a 200-mm telephoto lens, imaged mid-wavelength infrared light (3,000–5,000 nm), and had a temperature accuracy of ± 2°C and a maximum temperature saturation point for this study of 59.6°C. We experimented with this camera daily for short periods to try and characterize the surface temperatures of objects and animals flying in the flux field. The third type was a weatherproof surveillance camera (Model Q1614-E, Axis Communications, Lund, Sweden) that recorded in the visible wavelength range of light (390–700 nm; during the day) and had a wide dynamic range (WDR), enabling it to image both very bright conditions and dimmer adjacent objects. Two WDR cameras were run during all daytime hours and were usually equipped with variable-focus zoom lenses (Model Computar CS-mount 12.4–50 mm, CBC Americas Corp., Commack, New York, USA). We experimented with a range of different view areas and magnifications using the WDR cameras, including occasional imaging with a non-standard 90-mm zoom lens (Model LMVZ990IR, Kowa Company Ltd., Aichi, Japan). The fourth type of camera (Model KP-E500, Hitachi, Ltd., Tokyo, Japan) imaged in the near-infrared (NIR) wavelength portion of the spectrum (700–1,000 nm), was sensitive to very low-light conditions (0.00003 lux), and was used at night, supplemented with NIR light cast by a high-power illuminator (Model Mark 40, Luma Scientific, Warwick, Rhode Island, USA). The fifth type of camera (Model UVCorder, Oculus Photonics, Solvang, California, USA) imaged only in the ultraviolet (UV) spectrum (300–400 nm). We used the UV camera sporadically to image the tower and solar flux during the day with the aim of assessing whether the tower structure and surrounding light field may be perceived differently by animals that can see ultraviolet light (e.g., certain birds, insects, and bats). All cameras were placed approximately 12 m south of the base of each tower pointing upwards and more often than not imaged airspace to the west of the receiver (Fig 2). On 10–11 September, we experimented with mounting a WDR camera fitted with a 50-mm zoom lens on the west side of Tower 1 at ~38 m AGL; this camera pointed toward the southwest corner of the catwalk ~100 m AGL and imaged a small area of airspace a few meters south of the receiver. Camera resolutions, view areas, and minimum pixel sizes of detectable signals at relevant distances near the solar towers for the TS, SGT, and WDR cameras are provided in S1 Table. Cameras recorded imagery to on-board digital storage cards, digital video encoders (Model Q7411, Axis Communications, Lund, Sweden), or laptop computers with external portable hard drives using surveillance video software (Axis Camera Station, Axis Communications, Lund, Sweden). Cameras recorded imagery at a sampling rate of 30 frames per second (fps), with the exception of the NIR and WDR cameras, which sometimes recorded at 60 fps, and the SGT camera, which typically recorded at 10 fps.

Video imagery was copied to hard drives and backed up daily. All non-SGT video imagery was visually scanned at high speed (along key frames) using digital viewing software (VirtualDub 1.9.11, www.virtualdub.org). Next, motion analysis code developed for detecting bats at wind turbines (published as Supporting Information by [4]) was used to batch process the TS video imagery in its native format (avi) using matrix-based statistical software (Matlab with Image Processing Toolbox, Mathworks, Natick, Massachusetts, USA). This process flagged files containing targets of interest, thereby facilitating review of collected imagery. We visually reviewed all video sequences of interest noted during manual scanning and all those identified by the motion analysis software, then characterized events involving birds, bats, and insects, verifying the identity of objects detected by the TS with simultaneous imagery from the other cameras. We attempted to visually identify birds and insects in the WDR video imagery to the lowest taxonomic grouping possible. Select portions of SGT imagery were visually screened in support of observations from other cameras in order to show examples of thermal heating. Temperatures of targets were determined using proprietary software (FLIR Examine IR, v1.40.0, FLIR Systems, Billerica, Massachusetts, USA).

### Insect trapping

**Fig 4:**
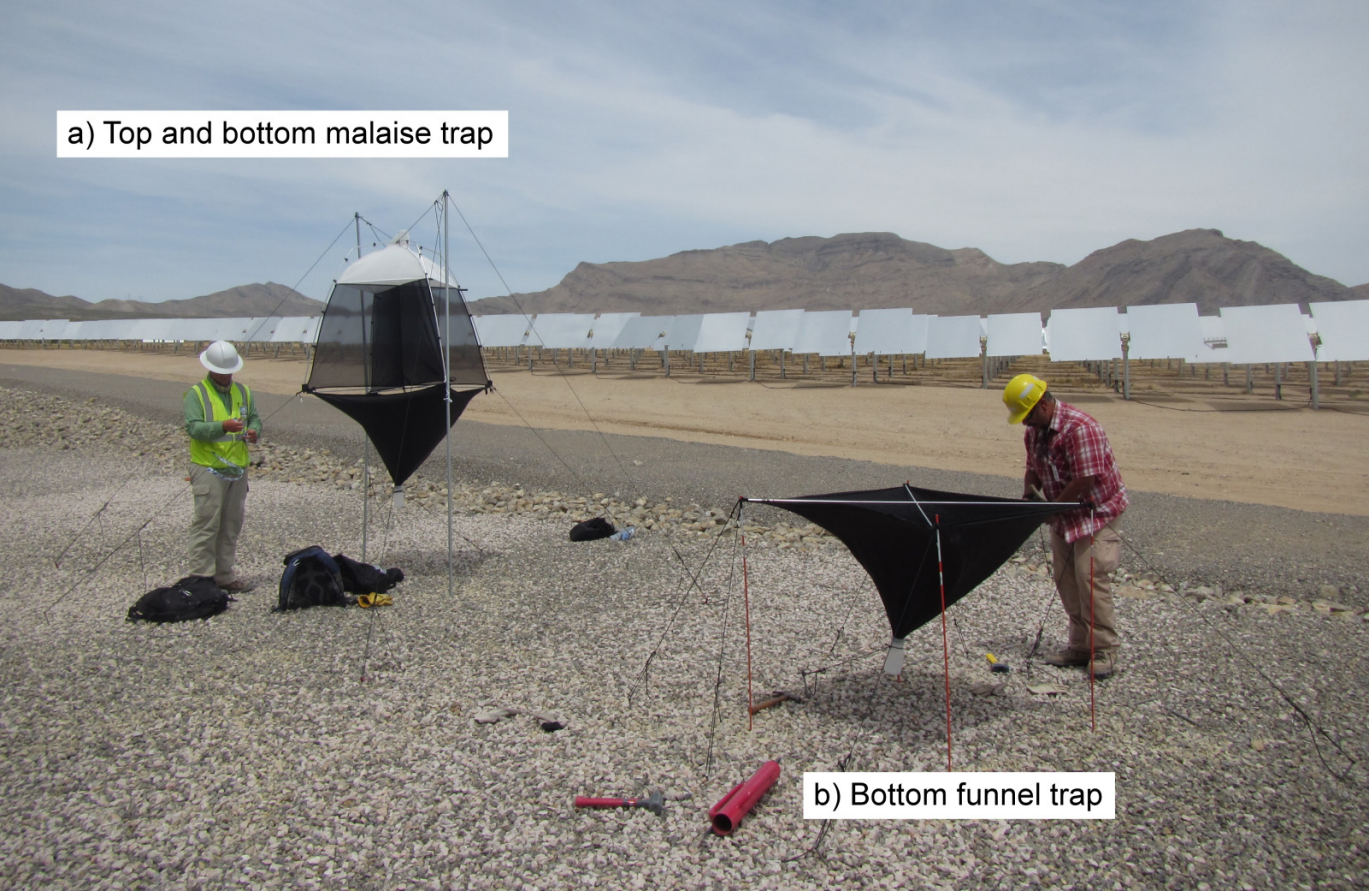
Setting up insect traps near a solar tower. Showing: A) complete malaise trap set up with “top” portion resembling a tent that is suspended between two 3-m poles and attached funnel below; B) funnel trap only that is suspended above ground with 1-m poles affixed to each corner. Photo by Paul Cryan, USGS.

We evaluated the feasibility of monitoring insect presence near solar towers using malaise traps. These two-part traps (Fig 4) included a top portion comprised of two orthogonal ~0.8 m^2^ panels that was suspended from 3-m poles staked into the ground and supported by guy lines. The top portion, which looks like a tent (Fig 4A), collected flying insects that alighted on the mesh panels and crawled upwards into a collection container. The bottom portion of the trap, a square funnel 1.1 m on a side (Fig 4B), was affixed beneath the top portion and collected flying insects that hit the panels of the top and dropped downwards. We also deployed bottom portions only, affixed to 1-m poles at each corner, to collect dead insects falling from the airspace above.

During 14–22 May, 11 insect trapping stations were placed around Tower 3 and within the area between the tower and heliostat array, as well as within open areas directly under the tower (Fig 2). Traps were deployed continuously throughout the day and night and were checked and emptied approximately every 10–12 hours. Traps at five of the 11 stations included both top and bottom portions (Stations 2, 5, 7, 9, and 10). Traps at Stations 1, 3, 4, 6, 8, and 11 only had bottom portions. In September, trap arrangement was the same as in May, but instead at Tower 2 (tower layouts are identical).

After collection, insects and related arthropods were taken to the USGS-Arid Lands Field Station on the University of New Mexico (UNM) campus, Albuquerque, where they were counted and visually identified to lowest taxonomic level, usually family but occasionally genus, guided by pertinent literature on insect identification [12, 13, 14, 15, 16, 17, 18, 19, 20, 21]. Reference specimens were examined at the Museum of Southwestern Biology, UNM, and some identifications were aided by consultation with resident entomologists. We generally followed the insect taxonomy of Triplehorn and Johnson [22], with the exception of recognizing the Family Lygaeidae [15].

## Results

### Radar monitoring

During the May and September site visits the radar ran continuously with occasional interruptions owing to loss of power, precipitation, or having to reposition the unit. The radar recorded 173 hours of data in May at Tower 3, and 72 and 79 hours at Towers 1 and 2, respectively, in September. Data acquisition rate of the radar was approximately 5.7 GB/hr. At any one location, the radar swept over a 7-km^2^ area which varied slightly depending on antenna elevation. The maximum altitude at which a radar can detect aerial fauna is limited by a number of factors, including antenna elevation and sensitivity of the instrument. Antenna elevations were low (between 4.2° and 9.0°) during this study, because our focus was to monitor airspaces that included the solar tower (140-m height), its solar flux field, and surrounding airspace. Across all three locations, altitudes monitored ranged from 24 m to 275 m above ground level, depending on antenna elevation, beam width, and near-field clutter.

Clutter produced by anthropogenic structures or the local topography is a routine challenge faced by radar biological operations [23] and ISEGS was no exception. The solar towers produced concentrated clutter that, in their immediate vicinity, obscured movement of animals and caused additional rings of radar clutter to varying degrees depending on conditions at each sampling location (e.g., Fig 3). Similarly, heliostats generated clutter near the radar, often out to and sometimes exceeding 500 m range. Although solar tower clutter was relatively static, heliostats produced dynamic clutter where even slight changes in heliostat position could yield a considerably altered clutter field. Other anthropogenic structures including high-tension power lines and fence rows produced spotty clutter, and terrain caused considerable clutter to the west while monitoring at Tower 2. At Tower 1, precipitation on late 7 September cluttered data collection and halted radar operations altogether during 8 September.

The radar recorded 708,872 biological tracks in May and 428,207 tracks in September according to our own post-processing algorithms applied to the output from radR [8]. Tracks include those caused by birds, bats, and larger bodied insects such as dragonflies, some beetles, butterflies, and moths.

**Fig 5:**
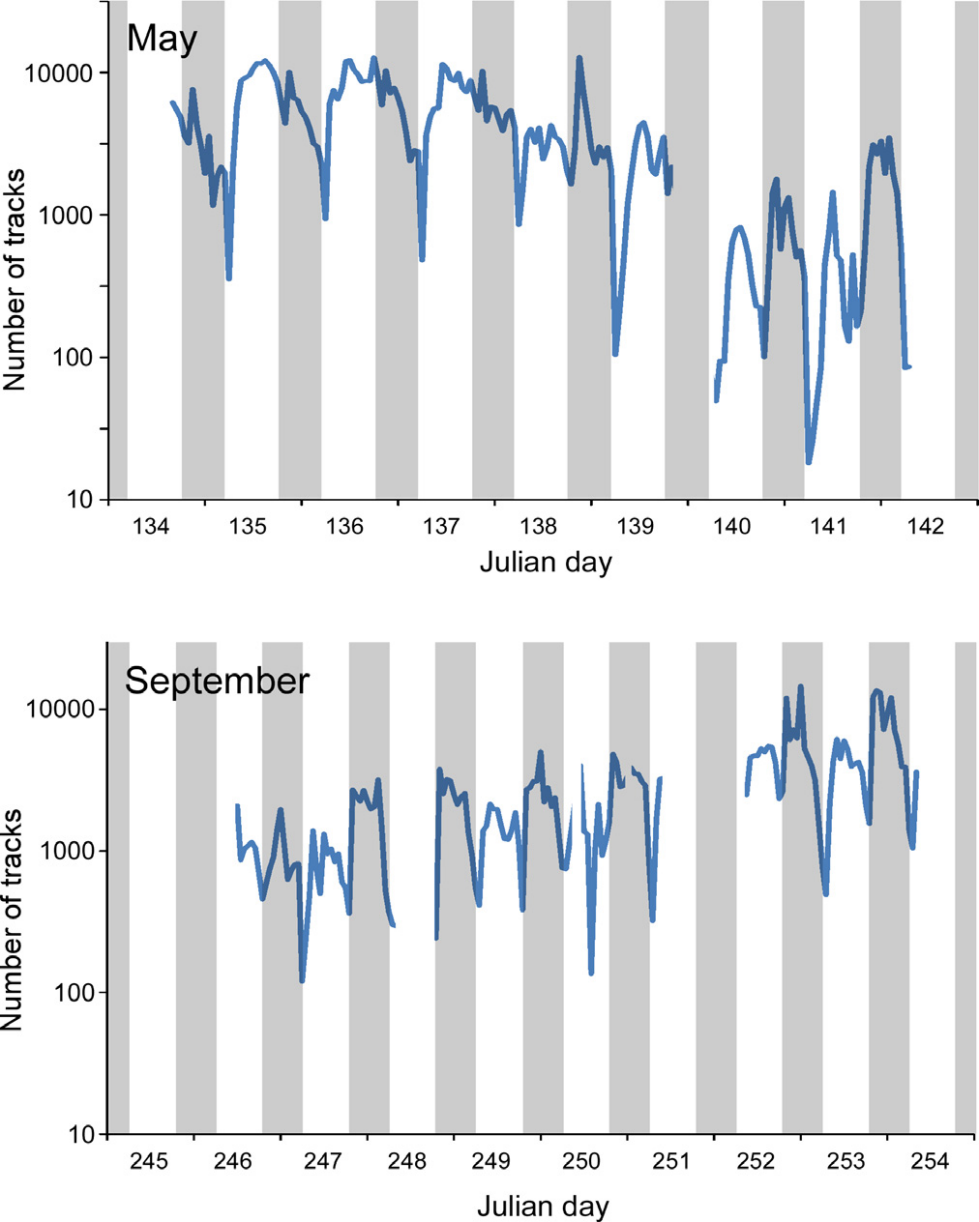
Number of radar biological target tracks. Julian day 134 refers to 14 May, 245 refers to 2 September, and shading differentiates night (gray) and day as determined by local civil sunrise and sunset.

**Fig 6:**
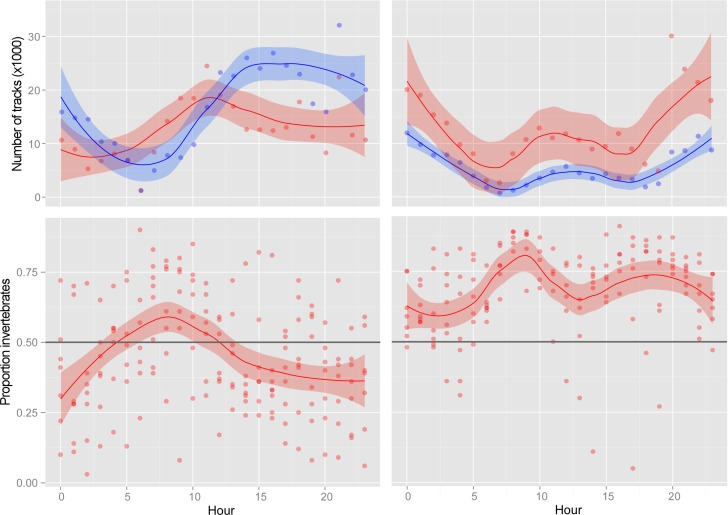
Variation in animal detections throughout the 24-hour day by radar in May (left) and September (right). Top) The number of tracks associated with vertebrate-like targets (blue) and invertebrate-like targets (red) cumulated for each hour throughout the 24-hour cycle from 14–22 May 2014 and 3–11 September 2014. Points show the number of tracks associated with each hour summed across all days, and the loess fit with 95% confidence limits captures trends in the data. Bottom) The corresponding relative proportion of invertebrates with loess fit and 95% confidence limits. A darker horizontal line indicates the 0.5 proportion level.

The intensity and timing of movement by birds and insects through the facility varied predictably, although the patterns of variation differed between May and September site visits and variation in animal use of the airspace was sometimes considerable (e.g., Fig 3; S1 Video). In May, the daily minimum abundance occurred after local sunrise at about 06:00 or 07:00 PDT, whereas in September the minimum abundance occurred either after sunrise (06:00 or 07:00 PDT) or around evening civil twilight (~19:00 PDT). Daily maximum abundance during both site visits was often more than an order of magnitude greater than the minimum but occurred at different times of the day; in May between 11:00 and 14:00 PDT and in September during late evening or around midnight (Fig 5). In May, targets classified as insects routinely comprised a larger proportion of aerial fauna than birds between about 04:00 and 12:00 PDT. Targets classified as birds, presumed to be mostly birds but likely also including some strong flying insects, were proportionately more abundant later in the day. During September, insects routinely outnumbered birds throughout the 24-hour day (Fig 6). A considerable increase in target abundance shortly after evening civil twilight was characteristic of daily abundance patterns during both May and September site visits.

Although solar flux was not measured directly, we used global irradiance, a measure of total energy from solar irradiance recorded by ISEGS as a proxy for the intensity of the flux field. During both site visits, global irradiance was highest between about 10:00 and 15:00 PDT (assuming no occultation of the sun by clouds), a period that overlapped the radar-determined transition from an insect- to bird-dominated airspace in May and was dominated by insects in September.

### Video monitoring

During the May site visit we operated the TS, SGT, WDR, NIR, and UV cameras in many different configurations (S1 Fig) and with the TS, WDR, and NIR cameras recorded a combined total of approximately 317 hours of surveillance imagery. During September, we again experimented with the TS, SGT, and two WDR cameras, but discontinued use of the NIR camera due to intractable illumination issues and poor image quality (S2 Video); in total we recorded 413 hours of surveillance imagery. We recorded approximately 1 hour of imagery with the UV camera during both visits, but this imagery was acquired for illustrative purposes only and not wildlife monitoring. The SGT camera was deployed in support of other video monitoring, usually for 20 to 120 minutes each day during mid-morning. Select portions of data from this camera were examined closely in conjunction with imagery gathered by the surveillance cameras. The SGT camera was not run continuously because its data rate was impractically high (up to 83 GB/hr), we had only rudimentary software to automate data processing of imagery, and the high cost of the camera makes it an unlikely candidate for routine, long-term, unattended monitoring. Imagery gathered with the UV camera indicated that the solar flux qualitatively appeared just as bright, or brighter, at short wavelengths of light (< 400 nm) as it did in the spectrum of light visible to humans (S1 Fig).

During the two visits, we were able to monitor the solar towers under a variety of conditions, both environmental (ranging from full sun to torrential rain) and operational (ranging from fluxing in full sun to non-operational in both stormy conditions and full sun), and observed highly variable frequencies of smoking objects in the flux of the tower. On some days smoking objects in the solar flux were sparse (S3 Video) and on other days they were frequent (S4 Video). Smoking objects were observed during approximately 56 hours of monitoring with the WDR cameras.

Thirty-seven hours of imagery gathered by the TS camera, usually equipped with the 35-mm lens, during the May visit and all of the TS imagery (213 hours) from the September visit were suitable for analysis using the automated processing software. Automated processing regularly detected aircraft, steam plumes released from the towers, clouds (the former all considered false positives), and occasional biological targets. Only rarely did objects seen smoking on WDR cameras simultaneously appear in TS imagery. The TS camera sporadically detected insects flying close (< 20 m) to the camera and, when equipped with the 35-mm lens, some large insects (e.g., dragonflies) flying at least as high as the catwalk surrounding the solar tower receiver (S5 Video), but not obviously higher. Otherwise, most flying animals imaged by the TS cameras were vertebrates (birds or bats). By reviewing simultaneous recordings from the TS and WDR cameras, it was possible to differentiate birds, bats, and large insects flying higher in the airspace (i.e., near the receiver) from smaller insects flying closer to the cameras; in general, close-flying insects appeared fuzzy and moved very rapidly through the TS video scenes compared to birds and bats, which were more sharply defined and transited scenes more slowly. Birds and bats moving as quickly through the scenes as insects were always close enough to the camera to be visually distinct (S2 Fig).

**Fig 7:**
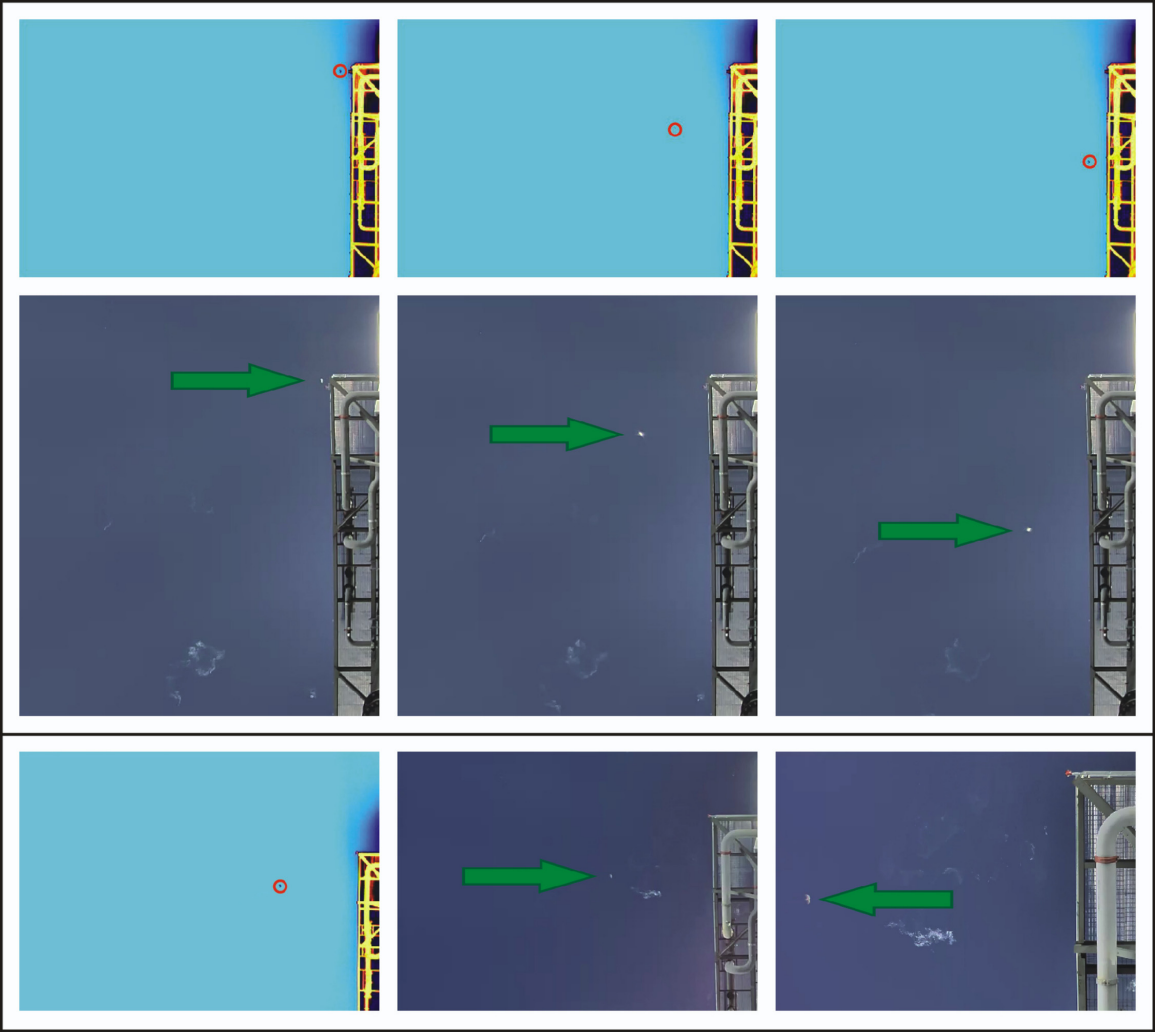
Birds in thermal video imagery and corresponding views from other surveillance cameras. Comparison of the thermal surveillance (TS) and wide dynamic range (WDR) cameras. Upper panel: still images from the TS (top row) and WDR (middle row) videos show a small bird near a solar tower over three consecutive seconds. Lower panel: still images from TS (left) and WDR far- (center) and near-view (right) videos showing the simultaneous detection of a medium-sized, falcon-like bird. Red circles in thermal frames show automatic target detection by processing software and green arrows show the corresponding bird in the WDR visual image.

Despite near-continuous operation of the TS camera with a 35-mm lens, smoking objects were rarely detected by it (exceptions were birds noted below), indicating most smoking objects were smaller than the 70–100 mm minimum-detectable-object size for that camera configuration (see S1 Table). Smoke did not have sufficient heat to be imaged by the TS camera. Smoking events were ephemeral and, to the extent our fields of view would allow, never during our study did we see or image any birds or insects flying far beyond the solar flux while still smoking (S6 Video). In conjunction with the TS imagery gathered, simultaneous WDR recordings and occasional corresponding SGT images showing wide and close-up scenes of the flux field and tower helped determine the size and identity of smoking objects flying near the tower (Fig 7). Most smoking objects observed during both May and September were too small to be accurately identified, even using telephoto lenses on the highest-definition cameras or with the WDR camera mounted on the tower. Only in a few cases could we identify the larger types of insects burning in the flux or falling from it past the cameras (S7 Video). At maximum zoom (90-mm telephoto lens) from the ground, the minimum detectable target size of the WDR camera was 4 mm at the bottom of the receiver and 6 mm near the top (S1 Table). At these same distances, the SGT camera imaged insects with minimum sizes of 7 mm and 10 mm, respectively. Close-up camera views of the solar flux (e.g., WDR camera with 90-mm lens, WDR camera on tower, SGT camera with 200-mm lens) often revealed far more small objects, presumed to be insects, than could be seen with wider views (S8 Video). Our attempt to closely image insects and objects in the flux by mounting a WDR camera half-way up the tower mostly failed. Vibration of the tower created motion in the imagery that negated the benefits of this closer view.

**Fig 8:**
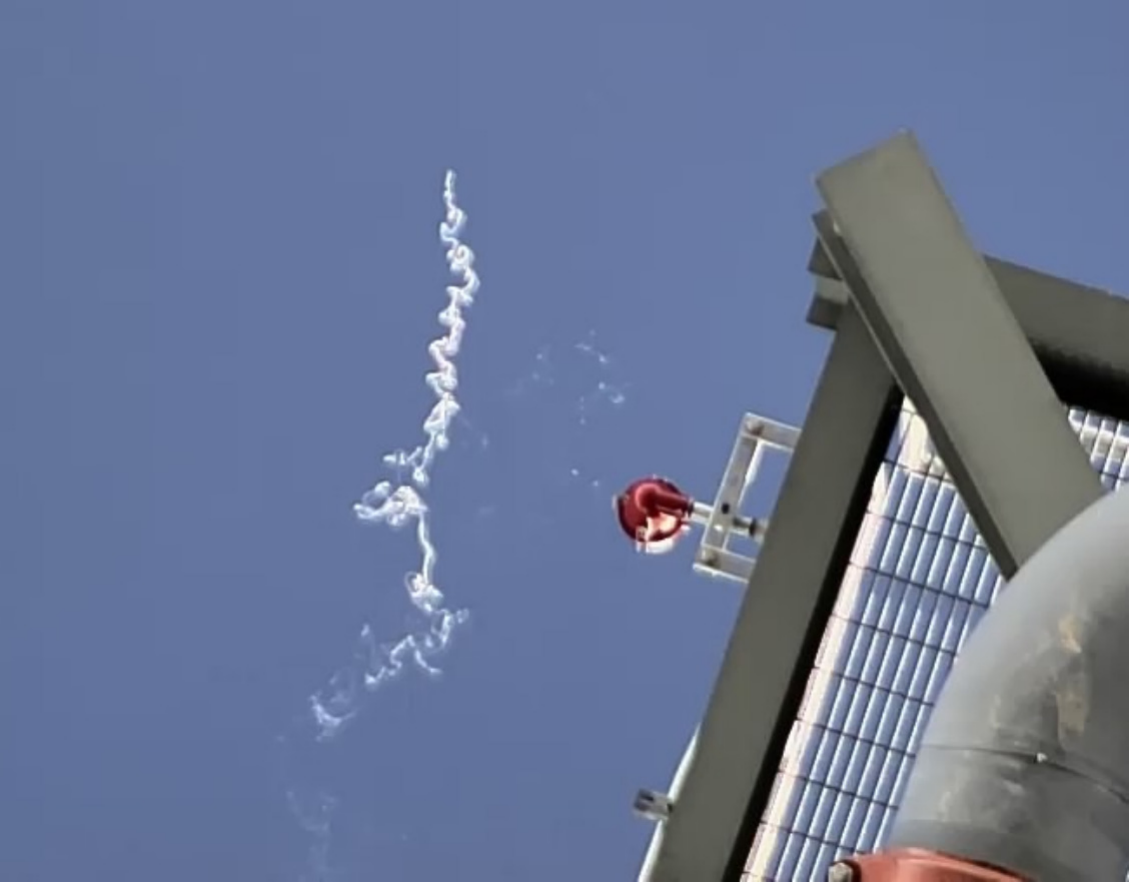
Erratic flight of insect burning in solar flux. Close-up view of what is presumed to be an insect smoking in the flux of a solar tower. Smoking objects often exhibit erratic flight trajectories, even in mostly windless conditions, suggesting powered flight. For perspective, the red object in the middle right of the scene is a light that measures approximately 12 cm in diameter and the smoking object is estimated to be 2–10 m above the light.

**Fig 9:**
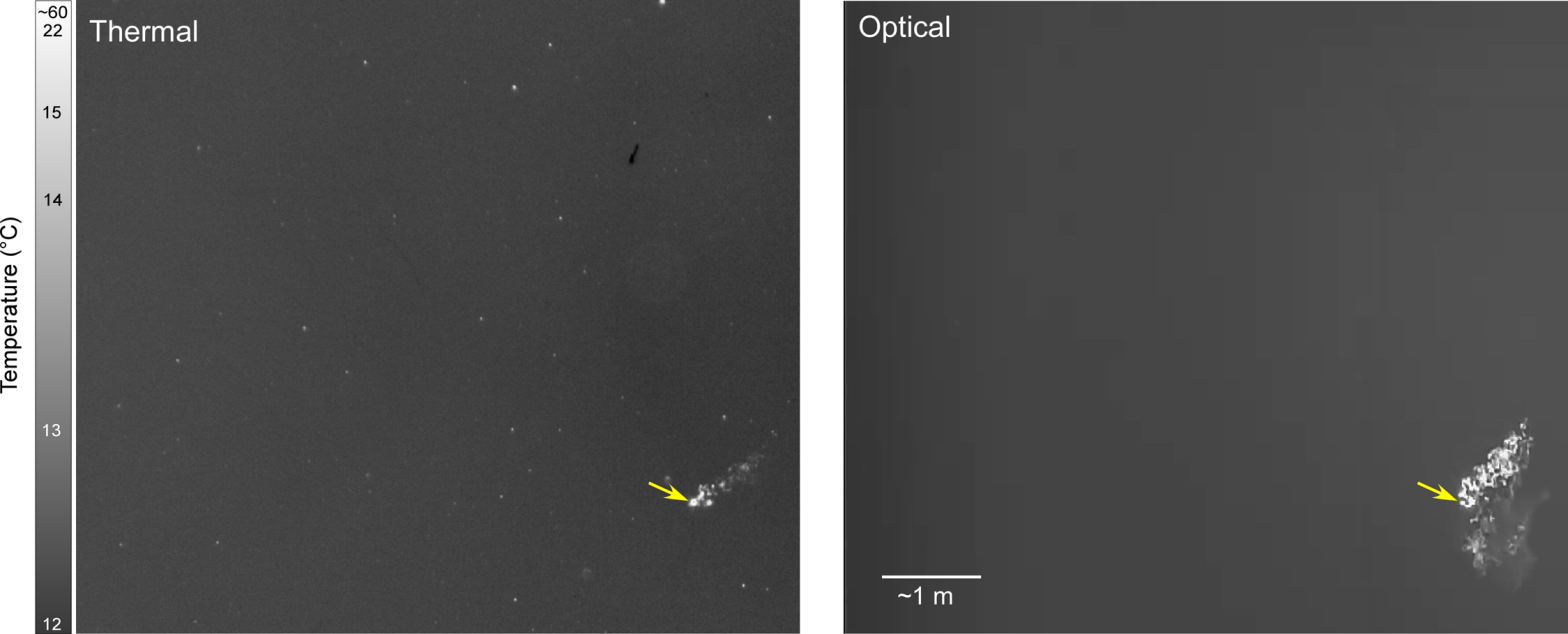
Burning insect imaged with different kinds of video cameras. Single frame from the scientific-grade thermal (SGT) camera (left) and wide dynamic range (WDR) camera (right) showing the same burning insect (arrow) in the Tower 1 flux field at 08:45:35 on 6 September 2014. The tower is just out of the field of view to the right. The images are adjusted to the same spatial scale, and the ~1 m spatial reference is an estimate based on approximate line-of-sight range to the insect. Bright points in the SGT image represent other probable insects in or near the flux field. A dark region in the upper-right of the thermal image is the ‘ghost’ of a target that was in the frame when a non-uniformity correction was performed. An animation from the SGT that concludes in this frame is available in S9 Video.

Video imaging regularly revealed complex trajectories of smoking objects (Fig 8), indicating powered flight or loss of it. Observations from the SGT camera also lend support to the assertion that most objects seen smoking in the flux field by WDR, but not TS, cameras were insects. Burning insects not imaged by the WDR camera were detected in the mid-infrared spectrum by the SGT camera (Fig 9). The SGT camera routinely detected insect heating above background temperatures, and the rate of heating varied considerably. In the instance depicted in Fig 9, the insect’s temperature increased exponentially over a period of about nine seconds; in the final three seconds, temperature increased by 40°C, to the point of saturation on the SGT camera (59.6°C; S3 Fig; S9 Video). Note that temperatures reported for targets detected by the SGT are most meaningful when considered relative to each other and not as absolute measures. Insects and to some extent birds flying through the flux field and recorded by this camera often act as small nearly isotropic radiators, and at the distances at which our observations were made (~100–136 m), spreading loss results in a considerable decrease in the amount of infrared energy reaching the camera for measurement.

We infrequently observed animals (including insects) flying near the tower receivers when flux was absent. However, we could not determine whether insects still occurred near the receiver when it was not fluxing, because the solar flux reflects brightly from objects within it, making them much more visible and detectable (typically as white flickering objects) when solar flux is present.

All events that we found involving birds produced clear visual signals in the TS imagery. A total of 37 birds or suspected birds were observed near the towers on video. Four of these events involving birds were found by visual scanning of video that could not be automatically processed, and the remainder were found by automated processing (S2 Table). Of these bird observations, five were made during the night and 32 were made during the day, with 29 of the daytime observations occurring when solar flux was present. Most birds flew above the fluxing tower without obvious signs of distress, sometimes while smaller objects smoked in the lower airspace, nearer to the receiver (S10 Video). Some of the birds appeared to flash (n = 9) and/or smoke (n = 5) during observation (S6 Video). We did not observe any evidence of birds completely incinerating in the flux and in all cases birds were still observable leaving the scene after smoking events (e.g., S6 Video). In one instance we detected two pigeon-like birds fly onto the deck just beneath the fluxing receiver and roost there for 14 minutes before flying away (S11 Video). Birds were usually identifiable to genus or family in the WDR imagery, but the imaging area necessary for consistent species identification (usually zoomed in) was much smaller than the wider views where detections were more easily made. During several detections, large birds were so high above the tower that they were only imaged with the TS camera and not the WDR cameras (even on maximum zoom; S12 Video). It was sometimes unclear whether larger birds flying above the tower were being affected by flux and smoking, or whether ‘video ghosting’ accounted for visual trails we occasionally observed behind birds in the recorded imagery (S13 Video). From the ground, it did not appear as though concentrated flux regularly occurred higher than the top of the towers. Automated processing of the TS imagery resulted in the regular detection of bats flying and foraging near the towers during most of the nights of monitoring (S14 Video), and these events were sometimes imaged with the NIR camera during the May visit (S2 Video). Visual high-speed scanning of videos from all non-SGT cameras did not result in any bird or bat detections missed by automated processing of the TS imagery, and many of the birds and bats detected by automated processing were not found during visual high-speed scanning.

The most well documented instance of a vertebrate encountering the flux field occurred on 5 September when a small bird was visually confirmed to enter the flux field and was simultaneously imaged by the SGT, TS, and WDR cameras. This bird exhibited thermal heating, although not in the same manner observed in many insects. Radiated temperature increased by 6.1°C in 0.4 seconds when the bird’s wings were closed. During one video frame captured when the bird’s wings were open, radiated temperature saturated the SGT camera at 59.6°C (S4 Fig). This bird transited the frame almost linearly at an estimated 12.6 m∙s^-1^; a considerably faster and more direct flight speed than we observed with most insects unaided by wind, including the insect illustrated in Fig 9 that traveled across the frame at an estimated 0.6 m∙s^-1^.

Our cameras did not incur any sensor damage (e.g., image burn-in) despite being pointed directly at tower receivers for entire days under full solar flux conditions. Both the TS and WDR cameras imaged through extreme rain events and returned to normal image quality within minutes after precipitation subsided.

### Insect trapping

In general, complete sampling at all traps began on either the 2^nd^ or 3^rd^ day after stations were deployed in May and September. Malaise traps were effective at passively collecting insects that were flying near the ground during the daytime and nighttime. A total of 1,922 arthropods were collected in May and September (S3 & S4 Tables). These arthropods represented nine orders and 39 families of insects, Collembola, as well as one family of spider. In total, 691 arthropods were collected during May at Tower 3, and 1,231 during September at Tower 2. Daytime sampling yielded more arthropods in May (n = 427) and September (n = 766) than nighttime sampling during those months, at 265 and 465, respectively (S3 & S4 Tables). After all traps were fully functional in May, the number of insects captured per day peaked at 153 on 17 May, then plummeted to a total of only 39 on 21 May. Samples collected during the day in September also varied, averaging about 62 per day from 5–7 September and peaking at an average of about 137 per day from 9–11 September. During the nighttime sampling in May, total catch peaked at 52 per night on 17 and 18 May, dropped to about 14 insects per night on 21 May, then rose to a total of 22 per night on 22 May. Total number of insects captured per night between 5 and 7 September ranged from 35 to 42, then ranged from 84 to 107 on 8 to 9 September, and gradually dropped to 43 on 11 September.

Among the daytime samples collected in May, flies (Diptera) and leafhoppers (Cicadellidae: Hemiptera) were the most abundant, whereas nighttime samples were dominated mostly by flies, but also included true bugs (Hemiptera), ants, bees, and wasps (Hymenoptera) in smaller quantities. Samples collected during the daytime in September were similar to those collected in May, with flies being the most common. However, other insects such as minute pirate bugs (Anthocoridae: Hemiptera), leafhoppers, and rove beetles (Staphylinidae: Coleoptera) were captured in equal or greater abundance on certain days. Most of the samples collected during the nighttime in September contained large numbers of flies. However, flies were less common, and generally represented by fewer specimens, than ants, wasps, and beetles in samples collected after 7 September.

Overall, most insects collected in traps during this study were small, with body lengths < 5 mm, but most had body lengths < 2 mm. Capture samples were dominated by ants, bees, wasps, and rove beetles, as well as flies that included gnats, midges, and mosquitoes. Captured insects longer than 10 mm were infrequent, but when encountered were comprised mostly of beetles, including a handsome yucca beetle (*Enoclerus spinolae*, Cleridae: Coleoptera). Another large and noteworthy insect captured was a single variegated meadowhawk dragonfly (*Sympetrum corruptum*, Libellulidae: Odonata).

We also opportunistically observed insects that had recently fallen to the ground around the towers. In May, several dead tarantula hawk wasps (*Pepsis* sp., Pomplidae: Hymenoptera), some true stink bugs (Pentatomidae: Hymenoptera), and an occasional butterfly that was similar in appearance to a Mormon metal mark (*Apodemia mormo*, Riodinidae: Lepidoptera) were found beneath Tower 3. Most of these burned insects were seen near the west and south sides of the tower, as well as in an open area between the north side of the tower and the ACC. In September, we frequently observed considerable numbers (e.g., >20 individuals per day) of dead dragonflies belonging to at least two families (e.g., Aeshnidae and Libellulidae) on the ground beneath Towers 1 and 2, as well as large coleopterans such as handsome yucca beetles.

Dead insects and debris accumulated on the ground in certain places around the towers, such as along the raised edges of the concrete pad near insect trap #1. At such accumulation points, a variety of insects that also share similar seasonal migratory patterns as dragonflies were observed. For example, we observed several ladybird beetles (Coccinellidae: Coleoptera), handsome yucca beetles, true stink bugs, seed bugs (Lygaeidae: Hemiptera), and leaf-footed bugs (Coreidae: Hemiptera) in the accumulated piles of insects and debris.

## Discussion

Aside from human observers, there are few well-established methods for detecting and observing animals flying near solar towers. Searching the ground around solar facilities provides information on some of the animals injured or killed from collisions, burns, or other causes [1]. Visual observations, unaided or under magnification (e.g., binoculars or spotting scope), are effective at observing the presence, identity, and behaviors of animals flying near solar towers [1]. However, observing solar towers with human observers can be labor intensive and expensive—automated methods have the potential to help overcome this challenge. During this study we identified certain remote-monitoring technologies that might allow for more efficient, long-term detection and observation of animals near operational solar towers under a wide variety of environmental and operational conditions.

### Radar monitoring

Portable surveillance radars are routinely used to characterize biological movements at spatial scales comparable to the land area of the ISEGS facility. The movements of large numbers of flying animals can be tracked over wide areas when radars operate continuously and algorithms automate post-processing. Although radars are less well suited to exploring detailed movements at small spatial scales (e.g., within several meters of the solar towers), their observations give a larger context to other technologies capable of monitoring these airspaces.

During both May and September site visits, radar documented relatively consistent patterns of abundance in aerial fauna. A lull in biological activity routinely occurred around 06:00 PDT followed by increasing activity as the day progressed. Such transitions are consistent with a shift from nocturnal migration to diurnal movements during migratory periods. In May the highest levels of biological activity detected by radar occurred at times of the day that coincided with the presence of an active flux field around the solar tower. Measured abundances of both insects and birds peaked during this time of day, which generally coincided with video observations of smoking objects in the solar flux at towers and generally higher catch rates of insects during the day. This contrasts with September observations when peak movements in both birds and insects tended to occur at night.

In contrast to May, the proportions of birds seldom exceeded those of insects during September. This may reflect differing uses of airspace between the taxa, especially during nocturnal hours, with birds possibly flying in greater numbers at altitudes above radar coverage (e.g. [24]). Alternatively, our brief site visit may have overlapped with a period of exceptional abundance in passage insects; indeed, the brevity of both site visits offers a greater potential for biased sampling than a longer study. Such potential sampling bias might also explain why September bird abundances were considerably lower than those during May when, all things being equal, summer reproduction on breeding grounds adds large numbers of juveniles to the migratory population. A concurrent but more continuous effort based on monitoring daytime mortality documented more birds at the site in fall than spring [2]; however this result may not be indicative of overall migration activity through the area which includes nocturnal migrants not at risk from solar flux.

During both site visits, a marked increase in the abundance of bird and insect targets occurred near evening civil twilight. Such behavior is typically associated with the onset of nocturnal migration during these times of the year. These abundances in the airspace are typically exceeded or at least maintained as night progresses, but this was only the case with September birds. In May abundances of both bird and insect targets dropped precipitously immediately following the twilight exodus. Perhaps this was the result of animals selecting and occupying airspaces above radar-monitored airspace during nocturnal flight. Brief but regular pulses of high abundance may also occur if the ISEGS facility was en route between foraging and roosting areas that are routinely visited by birds or insects.

Abundances or total track counts based on radar data should be considered indices of the magnitude of activity rather than absolute direct counts. Considerable overcounting was likely, because many flying animals may have entered and exited the radar’s beam more than once during their passage through the radar coverage area. This is expected in radar studies using low antenna elevations and a narrow beam width. Under these operating conditions, it is nearly impossible for tracking software to reliably identify targets repeatedly entering and exiting the beam as one and the same. High-elevation conical scanning can address this [25], but such scan strategies were not possible for this study and would have failed to detect animals flying at low altitudes (e.g., at altitudes equivalent to the solar tower receivers).

Radars are limited in their ability to resolve the taxonomy of the organisms they detect, both by the nature of the animals under observation and the sensing data used to make these discriminations. We expected a diversity of insects and small birds, primarily Passerines, would account for most of the flying animals moving through the Ivanpah Valley. When using surveillance radars, the most commonly applied method for differentiating between birds and insects is based on airspeed. We used a 7 m∙s^-1^ cutoff to distinguish bird (faster) from insect (slower) targets. No single threshold is widely accepted and typical airspeed values used for discriminating birds from insects range from 5 to 10 m∙s^-1^ [10, 11, 26, 27]. In principle, this method can be reliable provided wind profile data are accurate and the flying animals under study do not contain large numbers of insects capable of high-powered flight—usually this is not well known. Insect trapping at ISEGS revealed a diversity of small- to medium-sized insects, and relatively few strong fliers that could complicate an airspeed-based approach to radar target identification. However, these insects were captured near the ground and may represent species or relative abundances distinct from those aloft. By contrast, the solar flux fields at ISEGS in effect serve as aerial samplers, allowing us to know some of the invertebrate taxa moving through that airspace. We know from video and insects seen on the ground around the towers that some larger insects, particularly dragonflies, were common in the airspace over the Ivanpah Valley during site visits. Dragonflies are powerful fliers [27, 28] that could exceed airspeed thresholds typically used to screen for birds in radar track data. As such, our reference in this paper to “vertebrates” or “birds” as determined by radar observations may well include varying proportions of insects capable of sufficiently high-powered flight that were misclassified as birds.

The operational wavelength of the radar (~3 cm), among other variables, places constraints on the smallest target size detectable, a limitation filled by other technologies employed in this study. The “insect” classification was likely dominated by large and mid-sized insects, whereas smaller insects were largely untrackable but likely contributed to background clutter. However, ground-based insect traps and the SGT camera were able to gather data on this aerial plankton, which can be extremely abundant. We suspect these numerous small insects were only evident on the SGT by being at least partially illuminated by solar flux and reflecting thermal energy rather than radiating absorbed heat, because most did not exhibit erratic motion or noticeably combust (although combustion may not be detectable in small insects). Moreover, insects that were observed to smoke in the SGT field-of-view moved at similar rates to non-smoking targets and appeared to be present at approximately the same focal length (Fig 9).

The larger spatial scales (relative to cameras) and fundamental principles by which radars operate make it challenging to resolve detailed interactions between flying animals at the solar towers. Range, radar beam width, pulse length, digital sampling rate, and signal processing all interact to influence the spatial resolution of the radar. For example, during May the radar was positioned about 1,000 m from Tower 3. At that range, a 2.1° half-power radar beam yields a sample volume >36 m in diameter. Two animals within that space at the same range likely present as a single target. As with other tall anthropogenic structures, solar towers cause considerable clutter that can blind radar to the movements of animals in the immediate vicinity of that structure [4]. Solar towers also reflected of off-axis radio energy associated with radar antenna side-lobes which caused fixed-range arcs of measurable clutter. Despite these limitations, the broader context of animal movement that radar provided was not obtainable with any other method we examined.

### Video monitoring

Two of the video methods we tested show promise for detecting the presence of animals flying near solar towers, differentiating birds and bats from insects, and for observing quantities and behaviors of such groups. In particular, the ‘off-the-shelf’ availability and multi-spectral imaging capabilities of outdoor-ready video surveillance cameras (TS & WDR) make them particularly well suited for monitoring solar towers. Despite resolution limits that inhibited our ability to accurately detect and identify the smaller objects and animals (< 70–100 mm) with our TS camera equipped with a 35-mm lens, the combination of surveillance cameras and automated computer processing allowed continual monitoring and efficient detection of events of interest. These surveillance cameras functioned well through conditions of extreme weather and exposure that may have been impractical for human observers to endure. Another benefit of surveillance video is that a visual record of events can be acquired and analyzed repeatedly. This may be an important criterion when determining appropriate research or monitoring approaches, particularly where limited and ephemeral visual observations can be hard to interpret or contextualize. Our observations revealed that all may not be as it appears high above the ground around solar towers and that advanced monitoring techniques like video recording help reduce observational ambiguity.

Puffs of smoke and other flashing objects moving in and through the flux around solar towers can be visually confusing. The combined observations of our TS camera, which only imaged burning objects >70 mm but never smoke, with those of the WDR cameras, which regularly imaged burning objects and associated smoke, helped determine that the vast majority of the smoking objects observed were very small in length and/or width. Repeated attempts to image these small smoking objects using the WDR camera with a powerful telephoto lens (minimum detectable target size 4–6 mm; S1 Table) failed to clearly reveal their identities, with the exception of occasional larger insects (e.g., dragonflies). More-limited observations of smoking objects imaged using the SGT camera with a powerful telephoto lens further indicated that most smoke in the flux was attributable to objects too small to accurately identify from a distance of >100 m with the technology we used. In general, the smoke created by a burning object can make it appear larger and more dynamic, and this was particularly true in the solar flux around solar towers. Billowing, brightly illuminated smoke moving through the flux around solar towers sometimes created the illusion of birds in flight. Further complicating matters, occasionally birds could be seen flying high above the tower and were visible only as small white objects in the background, while similarly small-looking objects (presumably insects) simultaneously burned and smoked in the visual foreground (S10 Video). Sometimes the visual trails of these distant birds and those of closer, smoking objects intersected and created the illusion of the birds disappearing in smoke. However, we never observed evidence indicating that birds were completely incinerated by the solar flux. Concurrently and continuously monitoring the airspace with WDR and TS cameras helped us avoid potential confusion associated with ambiguous visual observations.

Our collective observations indicate that most of the small objects observed smoking in the flux were insects. We were unable to visually confirm the identity of most smoking objects because of their small size. However, they regularly moved on trajectories against the wind and often became erratic and descended downward while smoke was emerging, the latter indicating the loss of wing function. We often saw freshly burned insects on the ground beneath towers with crumpled wings, empty thoraxes lacking posterior structure, and/or individuals with missing heads. Insects losing flight control while smoke vents from the posterior or anterior regions of their thoraxes may explain erratic, often spinning trajectories observed on video with many smoking objects (Fig 8). Although we did not quantify the number of smoking objects imaged or their relative abundance on different sides of the tower in various conditions, we speculate that they may have been more prevalent on the leeward (downwind) side of the receiver. Insects often accumulate on the leeward sides of structures in wind [29] and quantifying the prevalence of smoking objects around different sides of a tower in relation to wind direction may help assess whether such objects are insects. It is unclear whether insects concentrate near solar towers when they are not fluxing. We rarely imaged any animals flying near a non-fluxing receiver, but this could be a detection bias associated with the WDR cameras being less likely to detect insects not illuminated by flux. Considering radar observations indicating the passage of large numbers of invertebrates in the airspace around the towers, it is possible that insects were present when the tower was not fluxing and that our cameras were not sensitive enough to image them. When we used WDR cameras with powerful zoom lenses or mounted them high on the tower we often saw large insects, such as dragonflies, flying in the flux and sometimes falling from it after burning, but these events only comprised a small proportion of the smoking events. Future use of more powerful camera lenses or placement of WDR cameras closer to the flux field (e.g., on the catwalk below the receiver) could help identify these smaller smoking objects. In general during approximately 08:00–14:00 PDT, we observed temporal correspondence between smoking objects in the flux, radar observations of peak numbers of insects in the airspace, and accumulations of burned insects observed on the ground around the towers. Collectively, these observations suggest that most smoking objects were insects.

Surveillance video cameras and automated computer processing of surveillance imagery were very effective at detecting the proportionally infrequent events we observed involving birds and bats flying near the towers. Even in cases when we suspect that very small birds were imaged (e.g., a possible hummingbird on 3 September), birds and bats created clear and detectable signals in the TS imagery. In no case during the two weeks of observation did we find evidence that birds were present near the tower and in range of the TS camera, yet not detected. Only a few birds were found in video imagery without automated processing, and all involved events in which they flew outside the view area of the TS camera or when the cameras were recording in a mode incompatible with the processing software. The automatic detection software found events lasting less than a second, but in most cases involving birds the software made multiple detections per event, spanning periods lasting from seconds to minutes. Although the maximum resolution of the TS camera we used may have been just above the edge of detectability for the smallest birds (e.g., hummingbirds) flying near the highest parts of the receiver, higher-resolution thermal surveillance cameras are now available that could improve range of detectability and imaging area. We were able to narrow down the identities of many birds initially detected with the TS camera by reviewing the simultaneous WDR imagery, but even at maximum zoom many were not imaged clearly enough to identify them to species. Higher-resolution WDR cameras could help overcome such challenges. Future improvements in the resolution and sensitivity of surveillance cameras will allow for monitoring bigger areas of airspace around solar towers with greater precision in identification.

Video monitoring also helped us understand the effects of reflected sunlight on animals flying as they were moving through the flux. We observed small birds burning in the flux and detected them with the TS, but were not able to acquire close-up imagery or temperature data for those events. The smaller sampling areas of zoomed cameras decrease the chances of imaging a region of airspace transited by a bird. The only events with birds that appeared to be burning involved small birds (e.g., warbler sized) flying within approximately 10–20 m of the receiver and in each case they appeared to descend downward after ignition. In one instance when we were able to image a bird moving through the flux with the surveillance cameras and the SGT camera, no visible burning or smoking occurred. However, the surface temperatures of that bird climbed quickly as it passed through the flux (S4 Fig). Although we do not believe long-term monitoring with the type of SGT camera we used is practical, such cameras could be used on a short-term, experimental basis in support of other detection methods as in this case, or to study the physiological stresses to flying animals imposed by radiation from the flux. The SGT imaged proportionally few events involving birds relative to the TS camera, because the attached 200 mm lens recorded a relatively small airspace (similar to the ground-based WRD camera with 50 mm lens) and the camera was only operating for short periods on some days.

Birds were regularly seen circling above the fluxing tower, where flux intensity should have been minimal (ISEGS, personal communication), and it was not always clear from the video imagery whether they were being affected by flux or not. We sometimes imaged what could have been either smoke emanating from feathers as the birds moved through the flux or an artifact of the video imaging process, known as ghosting (S11 Video). Future research could help answer this question by placing video surveillance cameras on top of the towers to monitor the airspace above. Some birds seemed to be regular visitors to the airspace above the fluxing towers. Ravens presumed to be resident to the solar tower facility during this study were occasionally detected circling above the fluxing towers with no apparent ill effects. We speculate that this behavior may reflect their opportunistic nature of scavenging insects from the ground around the towers that were burned in the solar flux. We also observed events in which birds quickly passed through the flux near the towers without obvious signs of distress or burning. For example, pigeon-like birds passed through the solar flux and roosted on the catwalk of the fully fluxing receiver while smoking objects occurred in the airspace nearby (S11 Video). In another event, a larger gull-like bird quickly passed near the face of the fluxing receiver (close enough to create a shadow) without visible smoke or burning (S15 Video). The surveillance cameras we tested were effective for detecting obvious burning and smoking, but they were not capable of detecting more subtle effects including how injury from contact with flux fields might impact animals away from the towers.

The TS and WDR surveillance cameras showed promise as being practical for long-term detection and observation efforts at solar towers; however, useful information was also obtained by the other cameras as well. The UV camera revealed that the flux and smoking objects within it are equally bright in the UV and visible spectra. Many birds and insects detect UV light [30, 31] and may perceive objects in the flux differently than humans. The NIR cameras were able to image bats and birds flying at night, but the visible lights on the tower at night interfered with the sensitivity of these cameras and limited their use. We did not observe bats or birds landing on or colliding with the towers at night. If the potential for collision events was a concern, as it was in the only prior study of birds at an early solar-tower facility [1], cameras capable of imaging at night such as the TS camera could be used to further investigate nocturnal bat and bird activity around towers.

### Insect trapping

The malaise and funnel traps were effective at capturing flying insects around the solar towers at heights ranging from the ground to approximately 3 m AGL. The number and types of insects collected varied with trap location, time of day, and season, as would be expected given the highly dynamic environment (S3 & S4 Tables). In addition to natural variability in the distribution and habitats occupied by insects, movement of air circulated by the ACC fans during the day, lighting on the solar towers and surrounding street lights at night, and possible low airflow (e.g., eddy) near some insect trapping stations also likely influenced the distribution of insects among the sites sampled.

In general, our insect traps caught mostly small insects and functioned well for obtaining a representative sample of insects present near the ground around solar towers. We could not determine whether assemblages of small insects similar to those we sampled occur higher around the towers in the flux. Our results suggest that the areas we sampled did not fully reflect the types and abundance of insects observed near the top of the solar towers, particularly during the day. From our observations, it appeared that insects of varying sizes tend to occur near the receivers when flux is present compared to those found in the surrounding ground-level airspaces where we put our traps. During both visits, we made occasional observations of larger insects (> 1-cm body length) dead on the ground below the tower that appeared to have been burned. Based on these observations, we identified tarantula hawk wasps (*Pepsis* sp.), butterflies, handsome yucca beetles, and true stink bugs. However, the most notable observations, relative to abundance and detectability, were several dragonfly genera (e.g., *Tramea*, *Sympetrum*, and *Anax*) belonging to the families Libellulidae and Aeshnidae during September. Many of these dragonflies were highly visible in the sky and on the ground, and in some instances were observed falling from the sky or descending away from the tower after being burned. The small area of our funnel traps did not effectively sample these types of larger insects falling from the flux above. For example, only part of one wing belonging to an unidentified dragonfly (Odonata) and one entire dragonfly were collected in the traps, whereas dozens of dragonflies were observed scattered on the ground near the towers in September. Additional methods of characterizing the types of insects active in different areas around these structures may be desirable considering the potential importance of understanding insect behaviors at solar towers.

Variability in the capture of insects among traps and over time reflected seasonal presence or phenology, abundance, and movement. Abundances of certain insects sometimes noticeably and rapidly increased, and these peaks were generally reflected by radar, video, and insect trapping. For example, the presence of alates (winged form of ants) often increases dramatically in deserts after rain [32]. We captured winged ants after a “100-year rain” event that occurred on 8 September, coincident with increases in the number of smoking objects seen on video in the flux after the storm. The high abundance of dragonflies observed during September compared to May suggested that this increased presence was potentially due to the movement of large numbers of individuals migrating through the area, as occurs in many species of Odonata. Like dragonflies, other insects (e.g., butterflies) show similar seasonal migratory behaviors. However, these migrations may not be concurrent or in the same direction; insect movements often are influenced by a number of variables, such as light intensity, temperature, wind speed and direction, as well as other weather-related factors that contribute to the timing and density of insects in the airspace [33]. Although we suspect that some of the insects we captured or observed were migrating through the area, many other species may be present year-round as residents of local habitats, either natural (e.g., handsome yucca beetle in Mojave Desert Scrub [6]) or anthropogenic (e.g., aquatic-associated species from the golf course to the east).

Insect trapping around the towers suggested potential origins of some of the arthropods burning in the solar flux. Given the location (Mojave Desert), we were surprised that aquatic and semi-aquatic insects were among those collected (e.g., backswimmers (*Notonecta* sp., Hemiptera), midges (Chironomidae, Diptera), mosquitoes (Culicidae, Diptera), certain species of dragonflies. The home ranges of many of these aquatic and semi-aquatic insects are limited by the availability of open water for reproduction, but many use flight to move into other areas when resources become limited. Although it is plausible that some of the aquatic insects we observed originated from a local ephemeral playa lake during September, their consistent presence before the rains and lake filling lead us to speculate that many originated from the aquatic habitats of the nearby golf course. Future studies could use more-comprehensive insect sampling techniques combined with chemical analysis of insect remains (e.g., stable isotopes) to help determine their origin and the influence local habitats may have on insect abundance.

It is possible that insects are attracted to the prominence of solar towers. Many insects, such as bees and wasps, flies, and moths and butterflies that were captured or observed near the solar towers during this study are known to be attracted to tall features in landscapes, such as tree tops and mountain peaks, which often serve as swarming or lekking sites (e.g., [34, 35]). Many of the insects captured or observed dead on the ground also tended to be taxa that may be attracted to light. Positive phototaxis is evident in a variety of insects, such as ants, bees, wasps, flies, dragonflies, moths, and butterflies, many of which we either trapped or saw around the towers. Phototaxis under natural conditions is known or thought to help insects orient themselves for movement and reproduction [36]. However, our study was not designed to test whether insects are attracted to the prominence or light of solar towers. These hypotheses could be tested through further research involving more comprehensive sampling and experimental manipulation with controls (e.g., insects sampled high on lit and unlit towers or on towers and similarly tall but less visually prominent structures).

A benefit of sampling with malaise traps like those used in this study is that they are effective at sampling small insects that might otherwise disperse in the wind or be difficult to find on the ground beneath towers. Malaise traps sample insects that are too small to be imaged on video or detected by radar and that may be important in determining the composition of objects burning in the flux field. Deploying malaise traps in a variety of locations within and outside the facility could provide a means of detecting insect activity around solar towers relative to weather, phenology, nighttime lighting, or other factors that may influence insect abundances at solar towers. It may also be possible to gather data that relate more specifically to the diversity and abundance of insects in the vicinity of solar flux by placing malaise traps at different heights along the tower and at increasing distances from towers and heliostat arrays.

## Conclusions

Interactions between flying animals and tall, human-made structures that reach into the aerosphere are difficult to predict and often require unique solutions that are only recognized after observing wildlife interactions. For example, the construction of tall buildings, communication towers, power transmission structures, and wind turbines resulted in unexpected bird and/or bat fatalities, often under difficult lighting conditions (e.g., darkness or clouds). It took several years to develop effective methods to observe how flying animals interacted with such structures and these delays likely inhibited the discovery of possible solutions. Therefore, developers of new technologies involving tall structures would benefit from understanding their effects on flying animals early in the development cycle to reduce potential adverse effects to wildlife and expensive retrofits to existing infrastructure. With the scale and brightness of solar power towers, new detection and observation techniques can help understand and potentially minimize the effects of this emerging technology on flying animals—our pilot research uncovered a few promising possibilities. Some of these methods could be further developed to effectively detect and observe wildlife flying near solar towers.

The radar observations revealed large quantities of aerial fauna moving through the airspace above ISEGS with bird and insect abundances tending to vary predictably through the 24-hour cycle. Although clutter obscured observations in close proximity to solar towers, radar was able to record overall use of the airspace by birds and larger insects. Radar’s inability to detect very small insects (aerial plankton) perhaps other than as background noise was confirmed by camera observations, specifically the SGT, and consistent with the insect assemblages sampled by ground-based traps. Radar’s ability to differentiate between birds and insects was complicated by the usual overlap between these taxa in airspeed, a metric commonly used in target discrimination. This is especially relevant at ISEGS since anecdotally observed insect mortality associated with flux fields indicates that dragonflies, strong fliers, occasionally were abundant in the airspace over the facility.

We evaluated the use of five different camera types to record interactions between flying animals and the solar towers at ISEGS. The UV, SGT, and NIR cameras were of limited utility for long-term research or monitoring efforts. The SGT played a useful supporting role in verifying observations of other sensors, giving insight into heating through temperature measures, and it was the only sensor to give some indication of the abundance of insects present in the vicinity of the tower. The combination of outdoor-ready TS and WDR surveillance cameras successfully captured imagery in the area of greatest solar flux near the receiver. These surveillance cameras, coupled with automated computer processing, allowed continuous remote monitoring of towers and efficient detection of events of interest. Furthermore, the cameras were successful in differentiating between birds/bats and insects; however, resolution of the TS camera limited the ability to accurately identify very small targets (< 70–100 mm) from the ground. Cameras equipped with higher magnification lenses or placing cameras higher on towers would likely provide the resolution necessary to identify smaller smoking objects and perhaps to identify birds to species. The use of TS and WDR cameras could provide a platform of sensors to efficiently monitor the airspace near solar towers and accurately identify targets within the solar flux field.

Malaise and funnel traps were successful in capturing flying insects at heights up to 3 m. The effectiveness of the traps would likely be increased by using larger traps and/or locating traps at different heights along the tower to better determine potential differences in insect types and abundance with altitude and proximity to the solar flux. The insect assemblages collected showed variations in the number and types of animals between different trap locations, time of day, and season. Insects observed likely originated from local habitats as well as migrated from farther locations. Although many of the insects collected and observed near the traps are known to be attracted to tall landscape features or exhibit positive phototaxis, a more experimental approach would be required to attribute the presence of these insects specifically to the solar towers or their flux fields. Although continuous monitoring using malaise and funnel traps near the ground could be useful in identifying insect activity related to flux production, there are likely more efficient ways of collecting or observing insects directly affected by solar flux.

## Supporting Information

S1 Fig Examples of scenes imaged using surveillance cameras at solar towers. Still images showing various scenes and recording schemes imaged using thermal surveillance (TS) cameras (first four rows), wide dynamic range (WDR) surveillance cameras (5th through 14th rows; for scale, the red light imaged in the 14th row is approximately 12 cm in diameter and can be seen at the corner of the catwalk surrounding the solar tower receiver in most camera views), near-infrared surveillance cameras (15th and 16th rows), and a camera that imaged only in the ultraviolet spectrum (17th through 20th rows).
(PDF)

S2 Fig Bird and bat imaged at close range with thermal surveillance camera. Still images of thermal surveillance video showing a large bird (top) and a bat (bottom) detected at night flying several meters above the camera.
(PDF)

S3 Fig Temperature of insect heating while flying in solar flux. Change in temperature over time of an insect entering the flux field at Tower 1 at 08:45:25 on 6 September 2014 as recorded on the SGT. Temperatures plateau at the camera’s saturation point of 59.6°C. This record corresponds with an animation presented as S9 Video. The last frame, and rightmost point in this figure, is depicted by the burning target in Fig 9.
(PDF)

S4 Fig Bird heating while flying through solar flux. Top: Six superimposed frames from the scientific-grade thermal imaging camera showing the track of a bird through the flux field on 5 September 2014. Temperature is indicated by color. Frame 5 from the left shows the bird with wings open and the camera saturated at 59.6°C. Bottom: Corresponding maximum temperature through time as the bird flew approximately south to north through the camera’s field of view. The series shows seconds only; the first frame occurred at 10:41:19.6.
(PDF)

S1 Table Specifications of video cameras used during study. Cameras listed by general type [including spectral range of sensor], model name, size of sensor array in pixels (px), and resolution (px/m), scene width and height, and minimum size of objects detected (i.e., necessary to fill a pixel), at two different distances relative to the bottom and top of the solar tower receiver (target distance). Model and lens configurations used for only a few hours during the study are marked with asterisks.
(PDF)

S2 Table List of all events in which birds were detected near solar towers. Bird detections in surveillance video imagery acquired during the spring and autumn site visits. Listed by sequential event number, date, time, whether clear evidence of burning in solar flux was observed (yes/no), descriptive comments on the event, and the types of surveillance cameras that detected the event (WDR = wide dynamic range, TS = thermal surveillance, SGT = scientific-grade thermal). Bold indicates that the event was found by automated computer processing of the TS imagery.
(PDF)

S3 Table Total number of insects collected during each daytime and nighttime sampling period in May 2014. Also showing stations with greatest and second greatest number of insects captured in parentheses. Samples collected in the top portions of the two-part malaise trap are labeled as “T” for top, whereas samples collected from bottom portions are labeled “B” for bottom. Multiple stations that shared an equal number of insects collected are listed together.
(PDF)

S4 Table Total number of insects collected during each daytime and nighttime sampling period in September 2014. Also showing stations with greatest and second greatest number of insects captured in parentheses. Samples collected in the top portions of the two-part malaise trap are labeled as “T” for top, whereas samples collected from bottom portions are labeled “B” for bottom. Multiple stations that shared an equal number of insects collected are listed together.
(PDF)

S1 Video Example of radar tracking flying animals. Video of portable radar output on 16 May 2014 from 10:55:57 to 10:59:59 PDT. Range rings in green denote 250 m intervals out to 1500 m. The radar tracks flying animals as yellow dots (most recent location) with blue trails (previous locations). The radar is located in the center of the frames and is surrounded by ground clutter caused primarily by the heliostat field. Tower 3 causes discernible clutter to the SSW and throughout a ~200° ring (faintly visible) at about 1000 m range. On this day, most radar detections are expected to be insects (Fig 6). The last frame is retained as the right panel in Fig 3.
(MP4)

S2 Video Bat flying at night imaged with near-infrared camera. Video sequences showing a bat flying past the receiver of a solar tower at night, approximately 100–140 m above ground level. First sequence shows imagery from near-infrared surveillance camera; the second shows same event imaged by thermal surveillance camera.
(MP4)

S3 Video Timelapse of solar tower with few smoking objects. Time lapse video imagery from a wide-dynamic range (WDR) surveillance camera showing approximately 11.5 hours of a fluxing solar tower on 20 May 2014. Mostly clouds and steam released from the top of the tower are imaged and few small, smoking insects were observed on this day.
(MP4)

S4 Video Timelapse of solar tower with frequent smoking objects. Time lapse video imagery from a wide-dynamic range (WDR) surveillance camera showing approximately 11 hours of a solar tower on 3 September 2014. The tower was operational from the start of the sequence until approximately noon, when it was taken offline. Small, visible and smoking objects that are presumed to be mostly insects occur frequently around the tower when flux is present, but then do not occur and/or are not observable after the tower is taken offline.
(MP4)

S5 Video Close-up of dragonfly near solar tower. Video sequences showing a dragonfly or other large 4-winged insect flying just above the upper catwalk of the solar tower. First sequence shows a close-up view imaged from a WDR camera mounted about half-way up the tower; the second sequence shows the same event from the same type of WDR camera positioned at ground level (briefly discernible looping in upper right corner near end of sequence).
(MP4)

S6 Video Small bird burning in solar flux. Video sequences showing simultaneous views from thermal surveillance camera (video inset to upper left) and wide-dynamic range camera of a bird flying close to the receiver of a fluxing solar tower. The bird appears to burn and then smokes within a few meters of the tower before exiting the scenes in the lower right near the end of the clips.
(MP4)

S7 Video Dragonfly in solar flux. Video sequences showing simultaneous views from thermal surveillance camera (video inset to upper left) and wide-dynamic range (WDR) camera of a large dragonfly flying close to the receiver of a fluxing solar tower. Dragonfly can be seen as small white speck hovering in upper right corner of WDR scene just above corner of catwalk, before it descends across scene from upper right to middle left, beginning about 28 seconds into the clip. The dragonfly is only faintly detected by the thermal camera in the early sequence when it is close to the fluxing receiver.
(MP4)

S8 Video Closeup timelapse of insects in solar flux. Time lapse of close-up video imagery from a wide-dynamic range surveillance camera with a telephoto lens (90 mm) showing approximately 1 hour of the airspace near a fluxing solar tower on 5 September 2014. For perspective, the red object in the lower left of the scene is a light that measures approximately 12 cm in diameter. Small bright objects presumed to be insects are seen and sometimes smoke while moving through the scene. Clouds and steam released from the top of the solar tower occasionally fill the background of the scene.
(MP4)

S9 Video Insect burning in solar flux. SGT camera video in real elapsed time of insects flying in and around the Tower 1 flux field on 6 September 2014 at 08:45:25 PDT. One of these insects begins burning part way through the animation. The tower is just out of the field of view to the right. Temperature in °C is to the right. Dark regions in the upper-right and lower-left of the animation are ‘ghosts’ of targets that were in the field-of-view when a non-uniformity correction was performed.
(MP4)

S10 Video Birds fly above solar flux and smaller smoking objects. Video imagery from WDR camera showing multiple objects (presumed to be small insects) smoking in the flux around the receiver of the solar tower while two large birds (Ravens) pass through the higher airspace above the tower and then circle above the flux to the northwest (left in video scene). This sequence demonstrates how it is difficult to assess the size of smoking objects without familiar objects for spatial reference.
(MP4)

S11 Video Birds roost on solar tower while solar flux is present. Video sequences showing simultaneous views from thermal surveillance camera (video inset to lower right) and wide-dynamic range (WDR) camera of two pigeon-like birds approaching and roosting on the catwalk around the fluxing receiver of a solar tower for approximately 14 minutes before departing briefly and then returning to roost again. First part of sequence is real-time until 11:31:17, roosting period is time lapsed until 11:45:19 when the sequence returns to real time. During the sequence, smoking insects, steam released from the top of the tower, clouds, and airplanes can be seen on the WDR and thermal cameras.
(MP4)

S12 Video High-flying, large bird detected only with thermal surveillance camera. Video sequences showing what was presumed to be a large bird flying high above the solar tower and detected by the thermal surveillance camera (upper left video inset), but not by the WDR cameras (zoomed view shown to right). The thermal camera sometimes detected large birds that were flying too high to be imaged by the WDR cameras.
(MP4)

S13 Video Bird above solar tower with smoke or video ghosting. Video sequences showing a large bird circling in the airspace to the west (left in video scene) of a solar tower with visible trails in video imagery. It is unclear whether these trails were caused by video ‘ghosting’ or were attributable to smoke caused by solar flux. First sequence shows a far view of the event followed by a shorter clip in which part of the event was captured on the same type of WDR camera with a zoom lens. Visible trails are less apparent in the close-up sequence.
(MP4)

S14 Video Bats foraging near solar tower at night. Thermal surveillance video of bats flying (presumably foraging) near the upper parts of a solar tower at night. Catwalk around southwest corner of tower receiver is visible to right of scene.
(MP4)

S15 Video Bird quickly flying past face of fluxing solar tower. Slow-motion video (one-quarter speed) of a large bird passing quickly near the south face of a fluxing solar tower receiver, passing within approximately 5 m and creating a shadow on the lit receiver. Smoking objects to the west (left) of the tower are smaller insects burning in the solar flux.
(MP4)

## References

[1] McCrary MD, McKernan RL, Schreiber RW, Wagner WD, Sciarrotta TC. Avian mortality at a solar energy power plant. J Field Ornithol. 1986;57: 135–141.

[2] HT Harvey & Associates. Ivanpah Solar Electric Generating System Avian & Bat Monitoring Plan—2013–2014 Annual Report Nipton, CA: HT Harvey & Associates.

[3] Taylor P. Bird deaths at Calif. power plant a PR nightmare for industry. Environment & Energy Publishing, 19 Jan 2015. Available: http://www.eenews.net/greenwire/stories/1060011853. Accessed 18 May 2015.

[4] Cryan PM, Gorresen PM, Hein CD, Schirmacher MR, Diehl R, Huso MM, et al Behavior of bats at wind turbines. Proceedings of the National Academy of Sciences. 2014;111: 15126–15131. 10.1073/pnas.1406672111 PMC4210316 25267628

[5] Larkin RP, Frase BA. Circular paths of birds flying near a broadcasting tower in cloud. J Comp Psychol. 1988;102: 90–93.

[6] Brown DE, editor. Biotic communities: southwestern United States and northwestern Mexico Salt Lake City, UT: University of Utah Press; 1994.

[7] Kirsch EM, Wellik MJ, Suarez M, Diehl RH, Lutes J, Woyczik W. et al. Observation of sandhill crane flight behavior in heavy fog. Wilson J Ornithol. In press.

[8] Taylor PD, Brzustowski JM, Matkovich C, Peckford ML, Wilson D. radR: an open-source platform for acquiring and analysing data on biological targets observed by surveillance radar. BMC Ecol. 2010;10: 22 doi: 10.1186/1472-6785-10-22 20977735 10.1186/1472-6785-10-22 PMC2988708

[9] Vaughn C. Birds and insects as radar targets: a review. Proceedings of the IEEE. 1985; 73: 205–227.

[10] Gauthreaux SA, Belser CG. Displays of bird movements on the WSR-88D: Patterns and quantification. Weather and Forecasting. 1998;13: 453–464.

[11] Larkin RP. Flight speeds observed with radar, a correction: slow "birds" are insects. Behav. Ecol. Sociobiol. 1991;29: 221–224.

[12] Arnett RH Jr. American insects: A handbook of insects north of Mexico, Second ed. Boca Raton, Florida, CRC Press; 2000.

[13] Arnett RH, Jr, Thomas MC. American beetles, Archostemata, Myxophaga, Adephaga, Polyphaga: Staphyliniformia. Vol. 1. CRC Press LLC, Boca Raton, Florida; 2001.

[14] Arnett RH Jr, Thomas MC, Skelley PE, Frank JH. American beetles, Polyphaga: Scarabaeoidea through Curculionoidea Vol. 2 CRC Press LLC, Boca Raton, Florida; 2002.

[15] Borror DJ, Triplehorn CA, Johnson NF. An introduction to the study of insects, 6 ed. Saunders College Publishing, Philadelphia, Pennsylvania; 1989.

[16] Borror DJ, and White RE. A field guide to insects, America north of Mexico Houghton Mifflin Company, Boston, Massachusetts; 1970.

[17] Evans AV, Hogue JN. Field Guide to California Beetles. Berkeley and Los Angeles, CA: University of California Press; 2006.

[18] LeBuhn G. Field Guide to the Common Bees of California: Including Bees of the Western United States University of California Press; 2013.

[19] Manolis T. Dragonflies and damselflies of California Berkeley and Los Angeles, CA: University of California Press; 2003.

[20] Powell JA, Hogue CL. California Insects Berkeley and Los Angeles, CA: University of California Press; 1979.

[21] White RE. A field guide to the beetles of North America Vol. 29 Houghton Mifflin Company, Boston, Massachusetts; 1983.

[22] Triplehorn CA, Johnson NF. Borror and DeLong's introduction to the study of insects, 7 ed. Thomson Brooks/Cole, Belmont, California; 2005.

[23] Alerstam T, Chapman JW, Backman J, Smith AD, Karlsson H, et al Convergent patterns of long-distance nocturnal migration in noctuid moths and passerine birds. Proceedings of the Royal Society London B; 2011; doi: 10.1098/rspb.2011.0058 10.1098/rspb.2011.0058 PMC3158935 21389024

[24] Larkin RP, Diehl RH. Radar techniques for wildlife research In: Silvy N, editor. Techniques for Wildlife Investigations and Management. 7 ed. Bethesda, Maryland: Wildlife Society; 2012 pp. 319–335.

[25] Chapman JW, Reynolds DR, Smith AD. Vertical-looking radar: a new tool for monitoring high-altitude insect migration. Bioscience. 2003;53: 503–511.

[26] Buler JJ, Dawson DK. Radar analysis of fall bird migration stopover sites in the northeastern U.S. Condor. 2014;116: 357–370.

[27] Wikelski M, Moskowitz D, Adelman JS, Cochran J, Wilcove DS, May ML. Simple rules guide dragonfly migration. Biol Let. 2006;2: 325–329. 17148394 10.1098/rsbl.2006.0487 PMC1686212

[28] Azuma A, Watanabe T. Flight performance of a dragonfly. J Exp Biol. 1988;137: 221–252.

[29] Lewis T, Dibley GC. Air movement near windbreaks and a hypothesis of the mechanism of the accumulation of airborne insects. Ann Appl Biol. 1970;66: 477–484.

[30] Lythgoe JN. The ecology of vision Clarendon Press, Oxford, England; 1979.

[31] Jacobs GH. Ultraviolet vision in vertebrates. Am Zool. 1992;32: 544–554.

[32] Johnson RA. Biogeography and community structure of North American seed-harvester ants. Annu Rev Entomol. 2001; 46:1–29. 11112162 10.1146/annurev.ento.46.1.1

[33] Johnson SJ. Insect migration in North America: synoptic-scale transport in a highly seasonal environment In. Drake VA, Gatehouse AG, editors. Insect Migration: tracking resources through space and time, Cambridge University Press; 1995 p. 31–66.

[34] Downes JA. The swarming and mating flight of diptera. Annu Rev Entomol. 1969;14: 271–298.

[35] Alcock J. Lek territoriality in the Tarantula Hawk Wasp *Hemipepsis ustulata* (Hymenoptera: Pompilidae). Behav Ecol Sociobiol. 1981;8: 309–317.

[36] Schwind R. 1991. Polarization vision in water insects and insects living on a moist substrate. J Comp Physiol A. 1991;169: 531–40.

