# Supplementary material for: Evaluating the Effectiveness of Wildlife Detection and Observation Technologies at a Solar Power Tower Facility
Source: PLoS One. 2016 Jul 27;11(7):e0158115. doi: 10.1371/journal.pone.0158115 (PMC4963080; doi:10.1371/journal.pone.0158115)

S1 Fig. Examples of scenes imaged using surveillance cameras at solar towers.

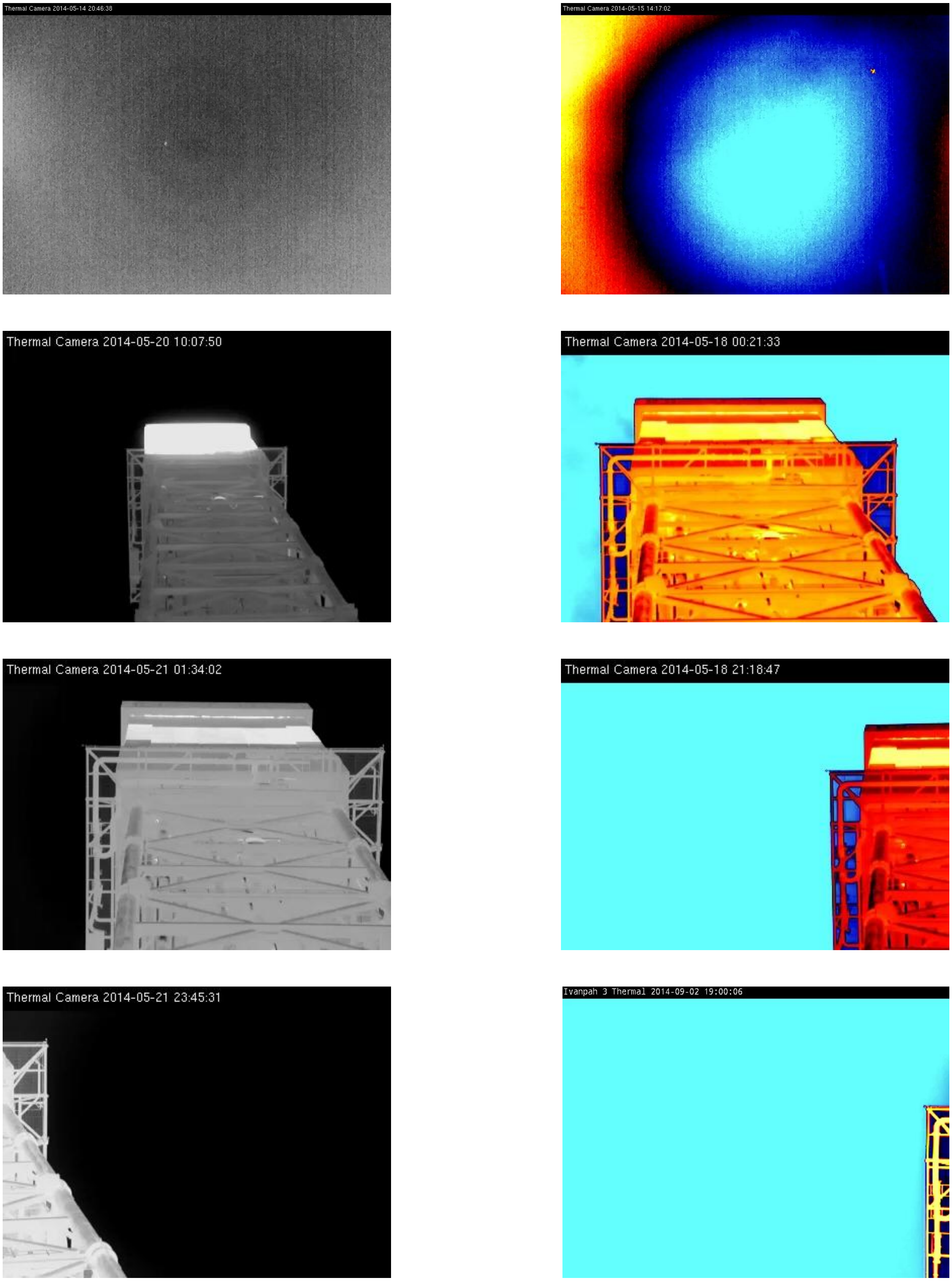

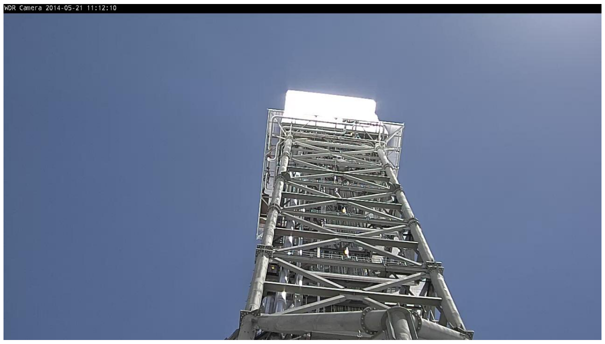
WDR Camera 2014-05-21 11:12:10

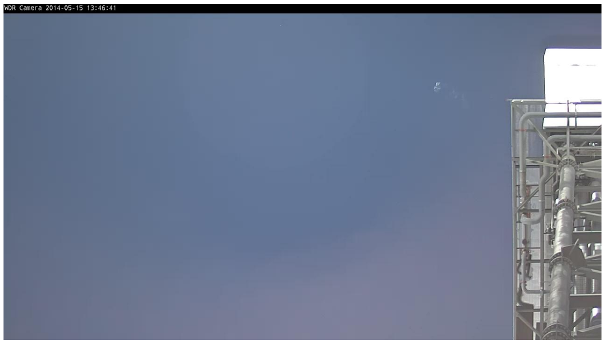
WDR Camera 2014-05-15 13:46:41

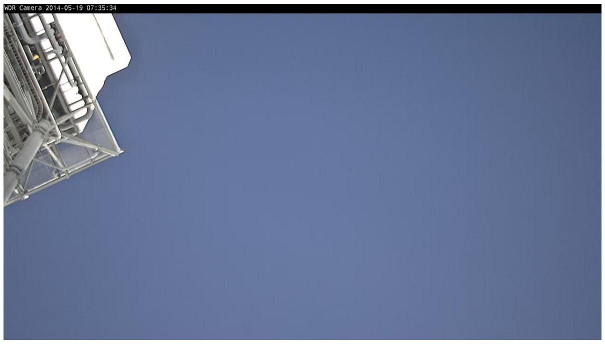
WDR Camera 2014-05-19 07:35:34

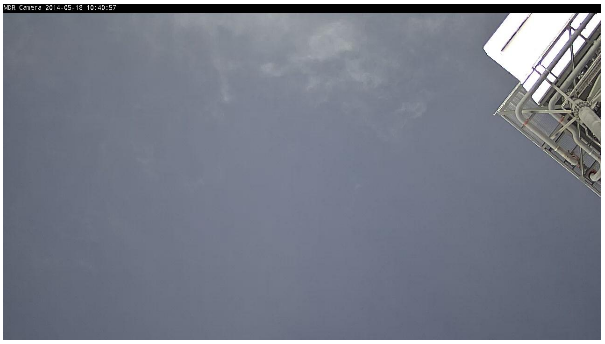
WDR Camera 2014-05-18 10:40:57

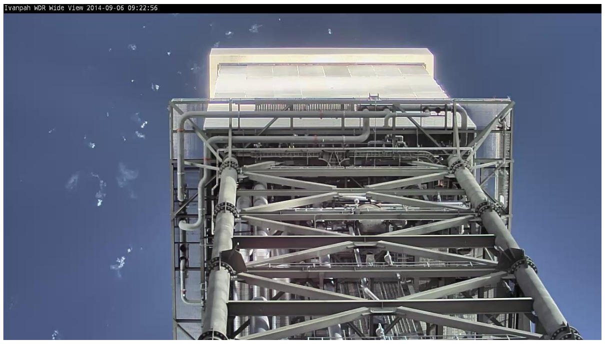
Ivanpah WDR Wide View 2014-09-06 09:22:56

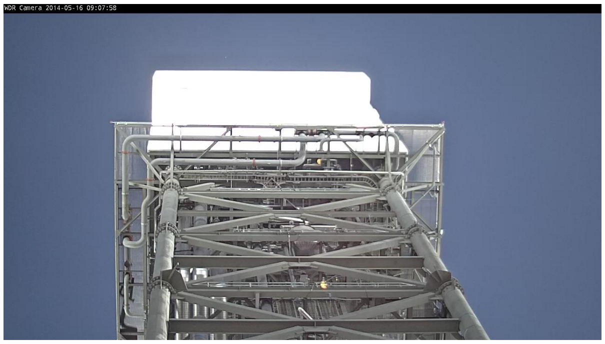
WDR Camera 2014-05-16 09:07:58

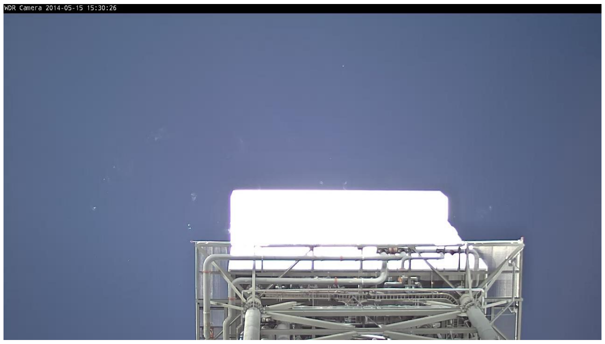
WDR Camera 2014-05-15 15:30:26

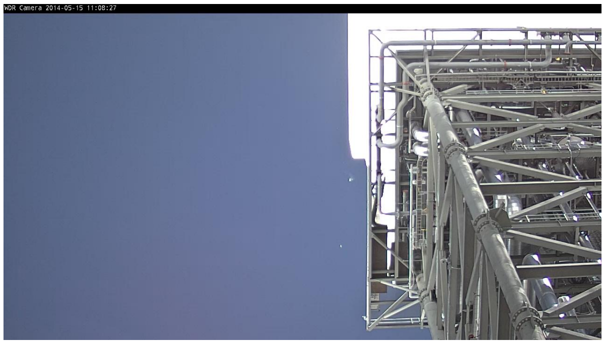
WDR Camera 2014-05-15 11:08:27

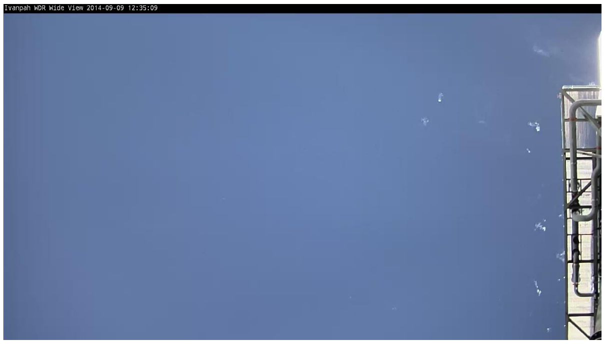
Ivanpah WDR Wide View 2014-09-09 12:35:09

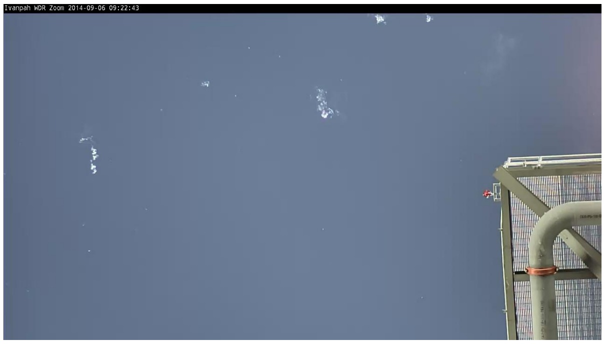
Ivanpah WDR Zoom 2014-09-06 09:22:43

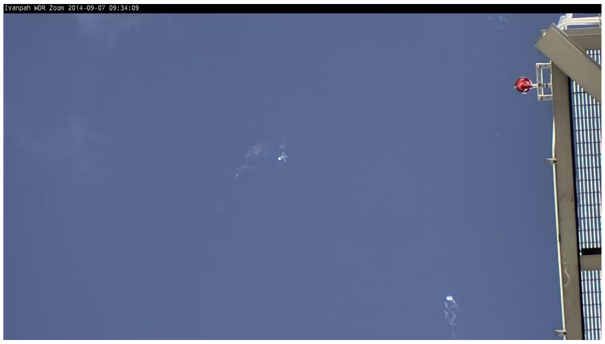
Ivanpah WDR Zoom 2014-09-07 09:34:09

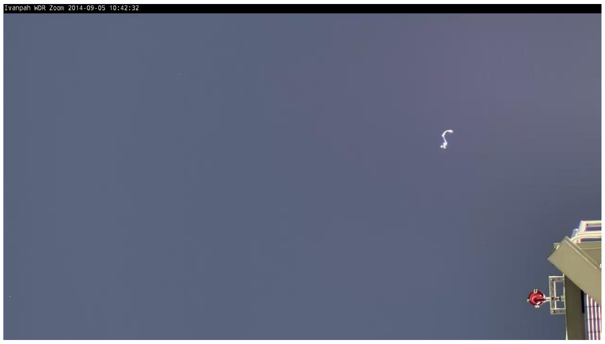
Ivanpah WDR Zoom 2014-09-05 10:42:32

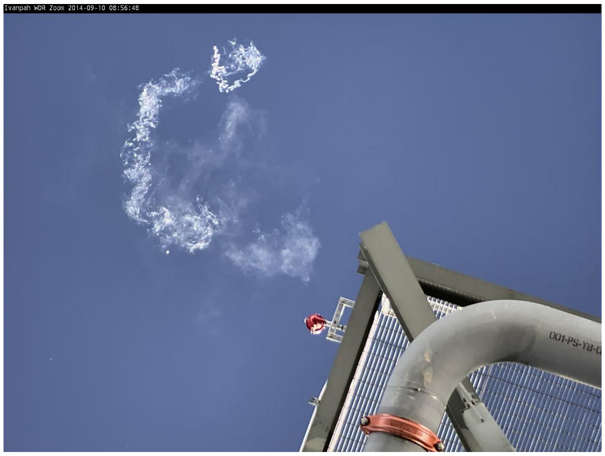
Ivanpah WDR Zoom 2014-09-10 08:56:48

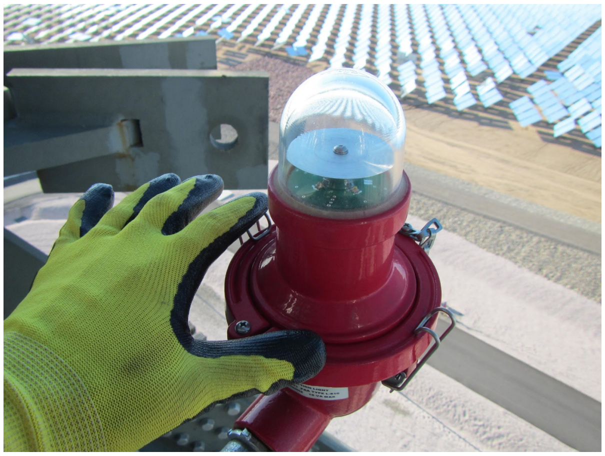

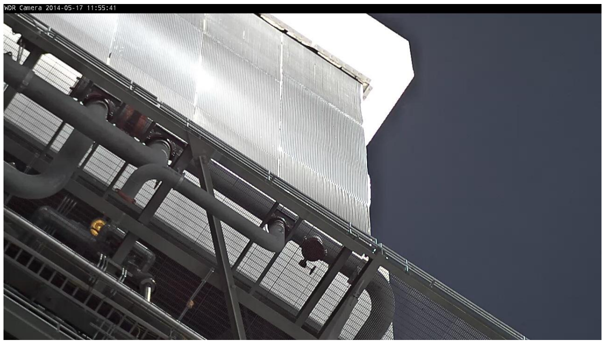
WDR Camera 2014-05-17 11:55:41

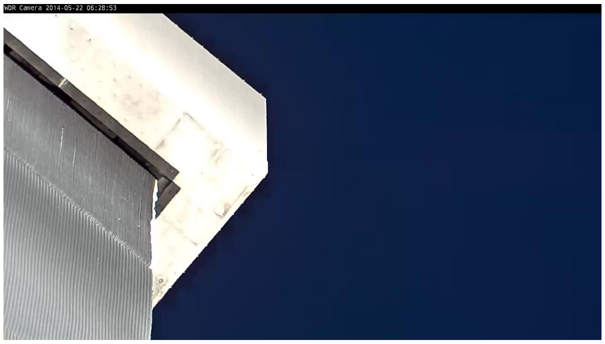
WDR Camera 2014-05-22 06:28:53

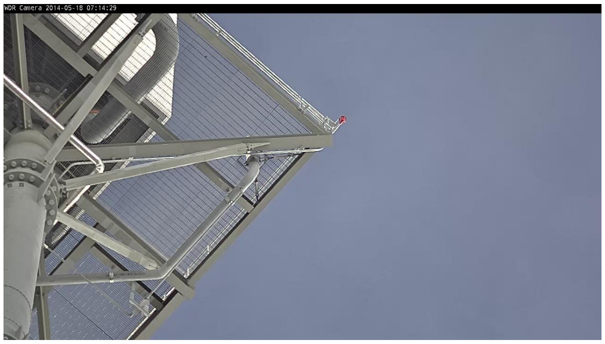
WDR Camera 2014-05-18 07:14:29

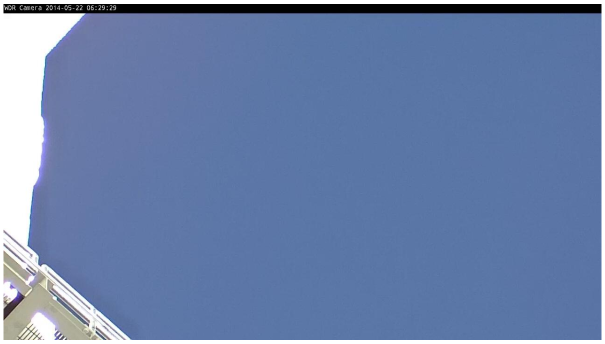
WDR Camera 2014-05-22 06:29:29

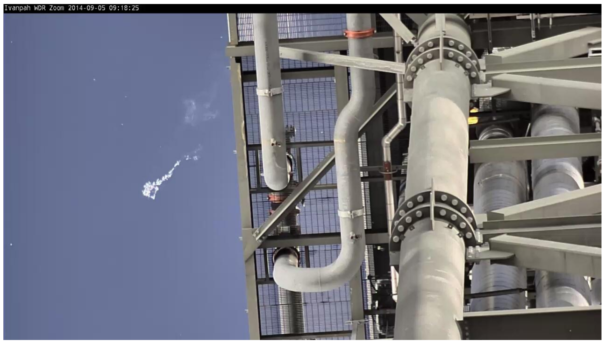
Ivanpah WDR Zoom 2014-09-05 09:18:25

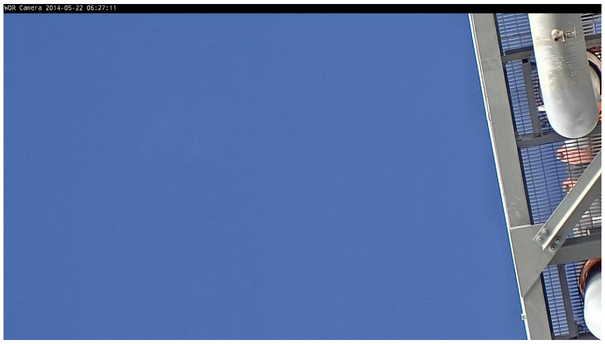
WDR Camera 2014-05-22 06:27:11

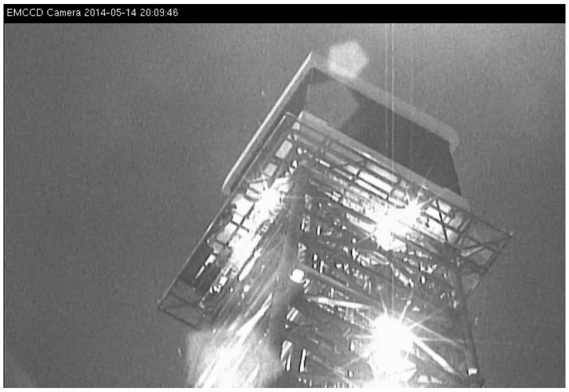
EMCCD Camera 2014-05-14 20:09:46

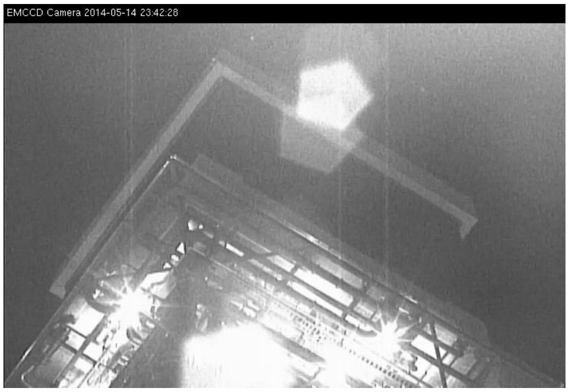
EMCCD Camera 2014-05-14 23:42:28

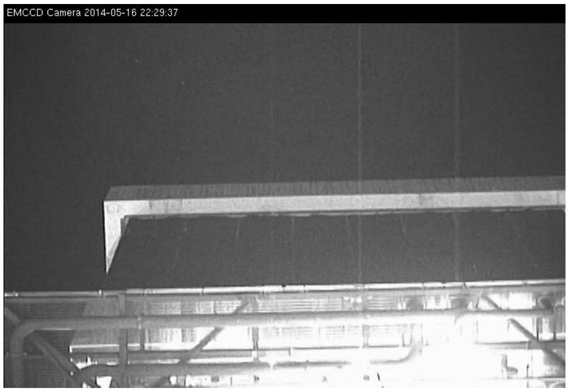
EMCCD Camera 2014-05-16 22:29:37

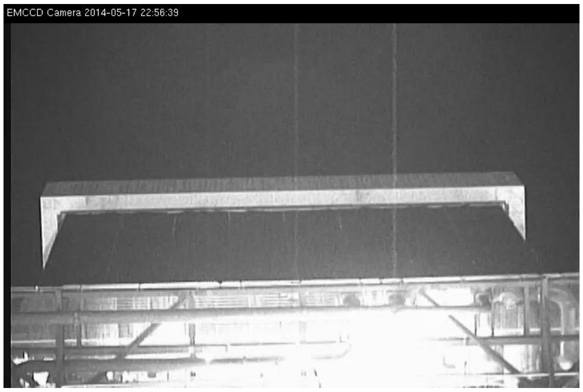
EMCCD Camera 2014-05-17 22:56:39

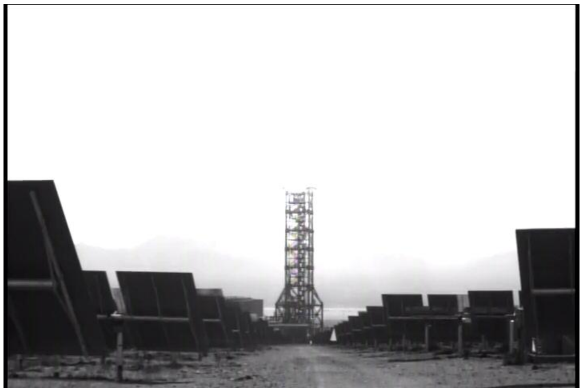

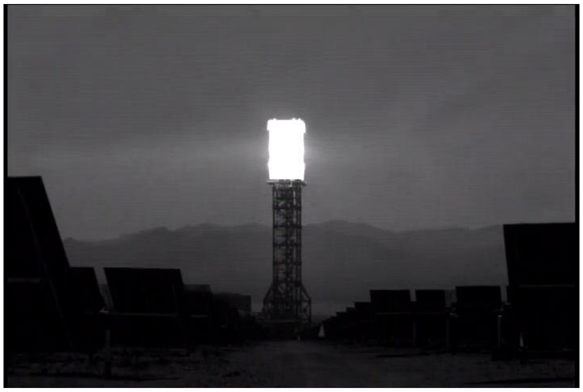

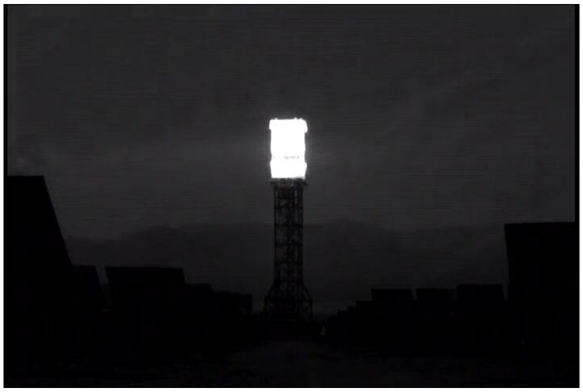

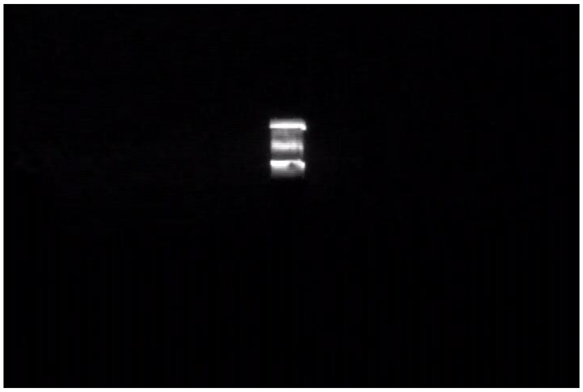

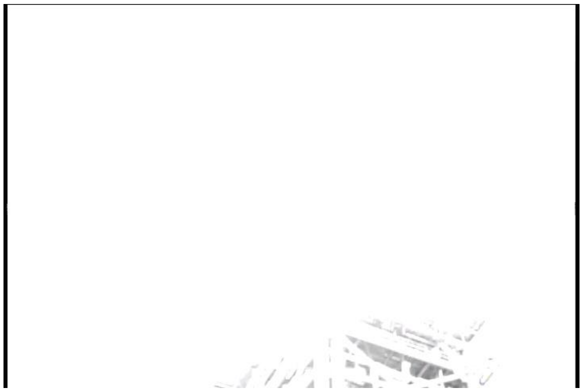

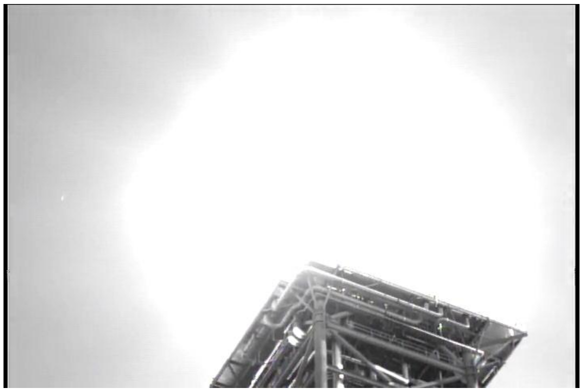

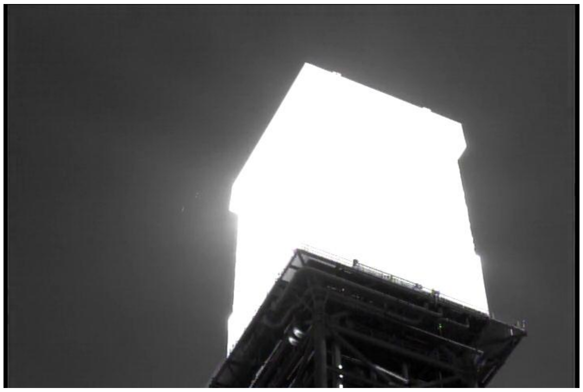

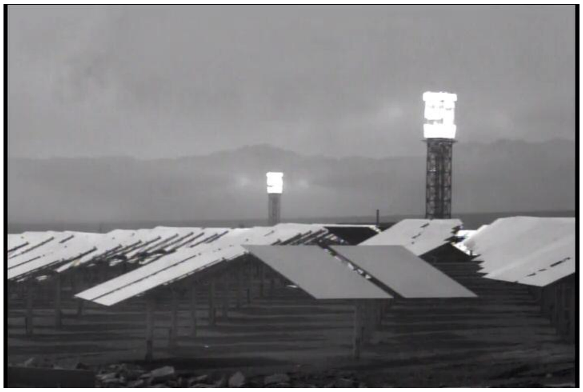

Supplement: S1 Fig — Still images showing various scenes and recording schemes imaged using thermal surveillance (TS) cameras (first four rows), wide dynamic range (WDR) surveillance cameras (5th through 14th rows; for scale, the red light imaged in the 14th row is approximately 12 cm in diameter and can be seen at the corner of the catwalk surrounding the solar tower receiver in most camera views), near-infrared surveillance cameras (15th and 16th rows), and a camera that imaged only in the ultraviolet spectrum (17th through 20th rows). (PDF) [file pone.0158115.s001.pdf]
