# Supplementary figures and images for: Evaluating the Effectiveness of Wildlife Detection and Observation Technologies at a Solar Power Tower Facility
Source: PLoS One. 2016 Jul 27;11(7):e0158115. doi: 10.1371/journal.pone.0158115 (PMC4963080; doi:10.1371/journal.pone.0158115)

**S2 Fig. Bird and bat imaged at close range with thermal surveillance camera.**

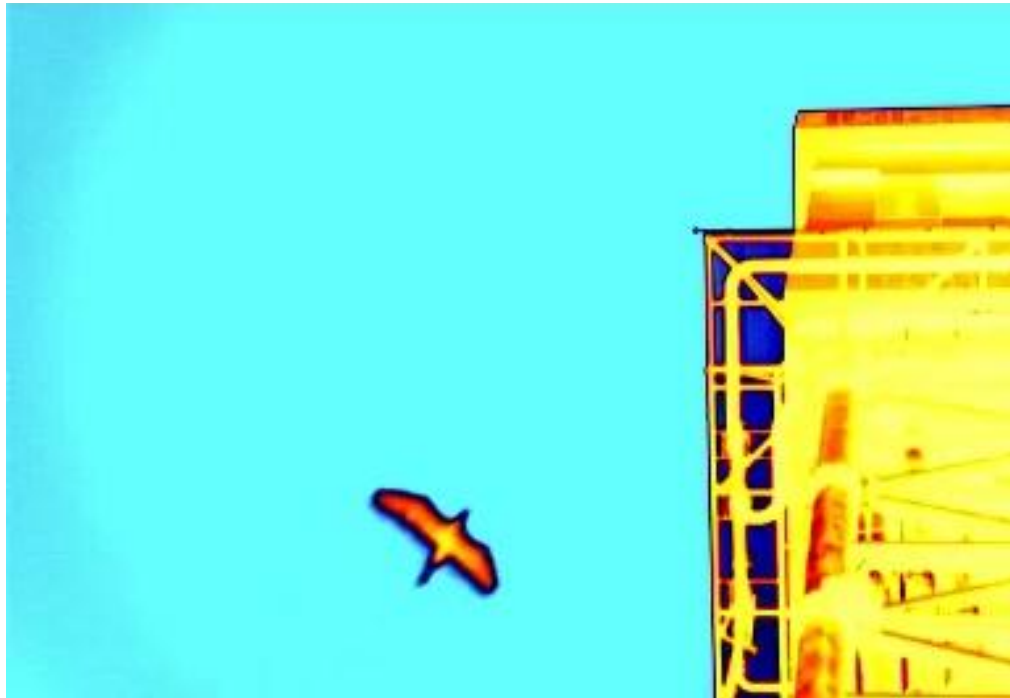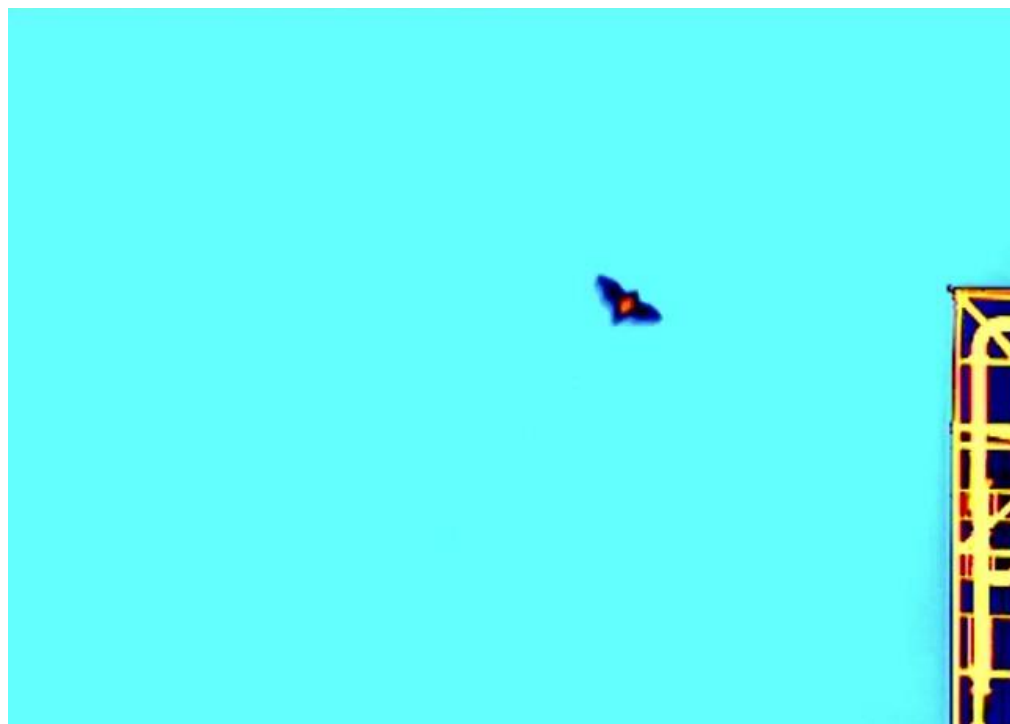

Supplement: S2 Fig — Still images of thermal surveillance video showing a large bird (top) and a bat (bottom) detected at night flying several meters above the camera. (PDF) [file pone.0158115.s002.pdf]

**S3 Fig. Temperature of insect heating while flying in solar flux.**

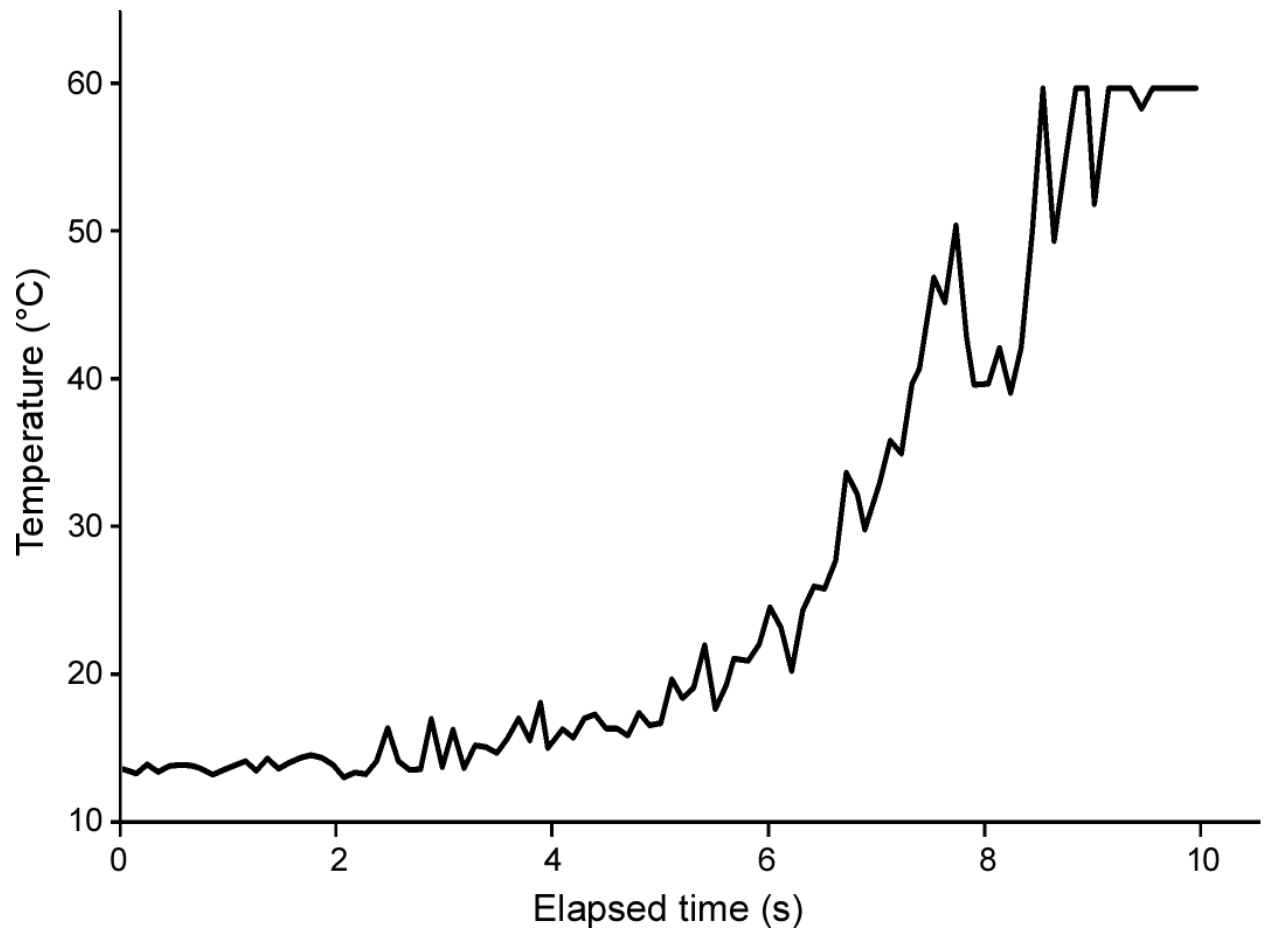

Supplement: S3 Fig — Change in temperature over time of an insect entering the flux field at Tower 1 at 08:45:25 on 6 September 2014 as recorded on the SGT. Temperatures plateau at the camera’s saturation point of 59.6°C. This record corresponds with an animation presented as S9 Video. The last frame, and rightmost point in this figure, is depicted by the burning target in Fig 9. (PDF) [file pone.0158115.s003.pdf]

S4 Fig. Bird heating while flying through solar flux.

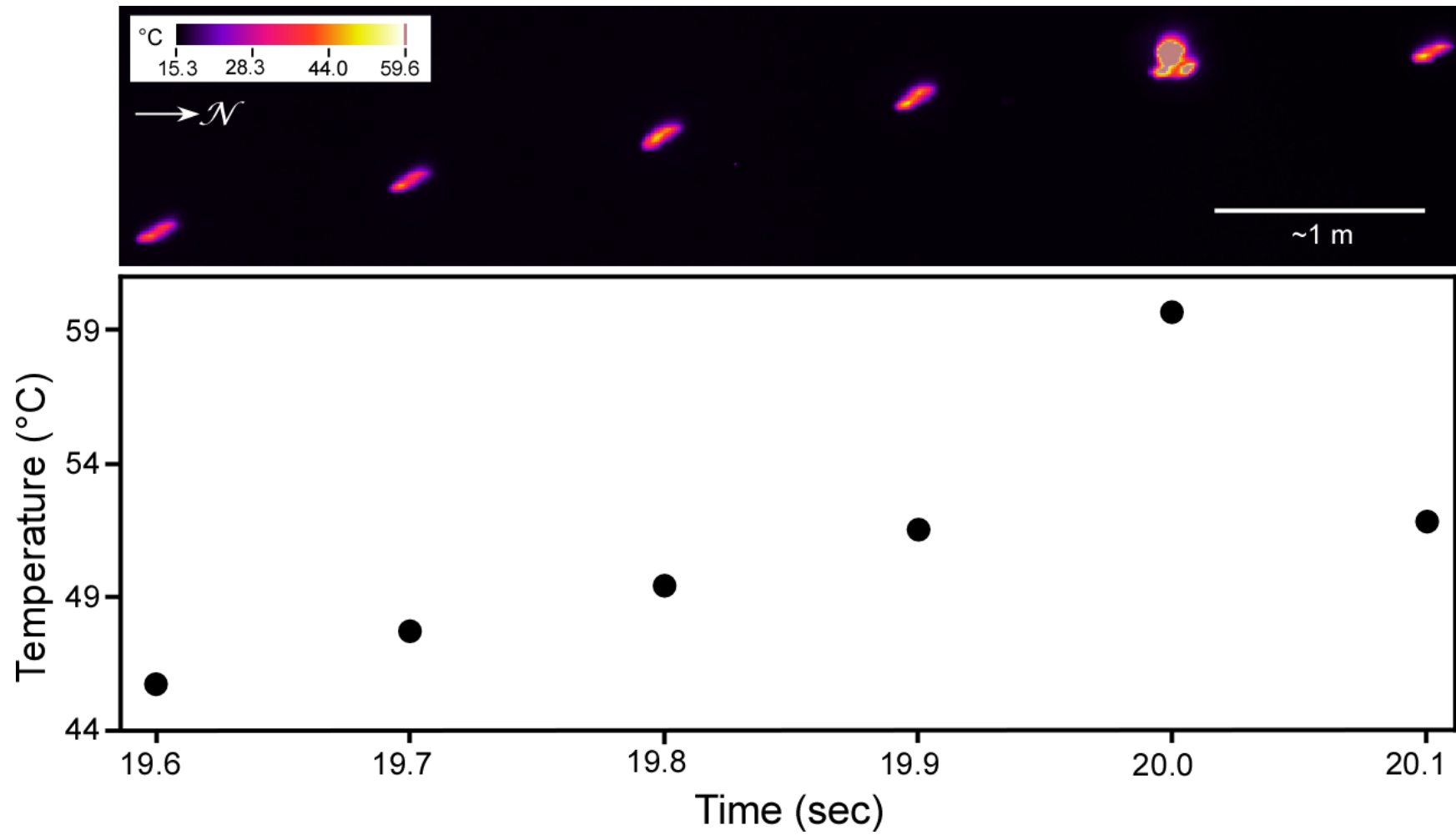

Supplement: S4 Fig — Top: Six superimposed frames from the scientific-grade thermal imaging camera showing the track of a bird through the flux field on 5 September 2014. Temperature is indicated by color. Frame 5 from the left shows the bird with wings open and the camera saturated at 59.6°C. Bottom: Corresponding maximum temperature through time as the bird flew approximately south to north through the camera’s field of view. The series shows seconds only; the first frame occurred at 10:41:19.6. (PDF) [file pone.0158115.s004.pdf]
