# Supplementary material for: Evaluating the Effectiveness of Wildlife Detection and Observation Technologies at a Solar Power Tower Facility
Source: PLoS One. 2016 Jul 27;11(7):e0158115. doi: 10.1371/journal.pone.0158115 (PMC4963080; doi:10.1371/journal.pone.0158115)
Supplement: S1 Table — Cameras listed by general type [including spectral range of sensor], model name, size of sensor array in pixels (px), and resolution (px/m), scene width and height, and minimum size of objects detected (i.e., necessary to fill a pixel), at two different distances relative to the bottom and top of the solar tower receiver (target distance). Model and lens configurations used for only a few hours during the study are marked with asterisks. (PDF) [file pone.0158115.s005.pdf]

S1 Table. Specifications of video cameras used during study.

Type [Spectral range]	Model and lens	Sensor array (px)	Target distance (m)	Resolution (px/m)	Width of scene (m)	Height of scene (m)	Min. object detected (cm)
Thermal Surveillance [9,000-14,000 nm]	Axis Q1921-E w/ 19-mm lens*	384x288	100<br>136	8<br>6	40.0<br>68.0	30<br>51.0	12.5<br>16.7
Thermal Surveillance [9,000-14,000 nm]	Axis Q1921-E w/ 35-mm lens	384x288	100<br>136	14<br>10	28.0<br>38.0	21.0<br>29.0	7.0<br>10.0
Scientific-grade thermal [3,000-5,000 nm]	FLIR SC8343HD w/ 200 mm lens*	1280x720	100<br>136	135<br>99	9.5<br>12.9	5.3<br>7.3	0.7<br>1.0
Wide dynamic range (on ground) [390-700 nm]	Axis Q1614-E w/ 50-mm lens	1280x720	100<br>136	131<br>96	10.0<br>13.0	5.5<br>7.5	0.8<br>1.0
Wide dynamic range (on ground) [390-700 nm]	Axis Q1614-E w/ 90-mm lens	1280x720	100<br>136	236<br>173	5.4<br>7.4	3.1<br>4.2	0.4<br>0.6
Wide dynamic range (on tower) [390-700 nm]	Axis Q1614-E w/ 50-mm lens	1280x720	63<br>99	208<br>132	6.2<br>9.7	4.6<br>7.3	0.5<br>0.8
Near-infrared [700-1,000 nm]	Hitachi KP-E500 w/ 50-mm lens*	640x480	100<br>136	89<br>65	5.4<br>7.4	7.2<br>9.8	1.1<br>1.5
Ultraviolet	Oculus Photonics	640x480	Variable	Variable	Variable	Variable	Variable

[300-400 nm]	UVCorder<br>w/ variable lens*						
