# Supplementary material for: Evaluating the Effectiveness of Wildlife Detection and Observation Technologies at a Solar Power Tower Facility
Source: PLoS One. 2016 Jul 27;11(7):e0158115. doi: 10.1371/journal.pone.0158115 (PMC4963080; doi:10.1371/journal.pone.0158115)
Supplement: S2 Table — Bird detections in surveillance video imagery acquired during the spring and autumn site visits. Listed by sequential event number, date, time, whether clear evidence of burning in solar flux was observed (yes/no), descriptive comments on the event, and the types of surveillance cameras that detected the event (WDR = wide dynamic range, TS = thermal surveillance, SGT = scientific-grade thermal). Bold indicates that the event was found by automated computer processing of the TS imagery. (PDF) [file pone.0158115.s006.pdf]

S2 Table. List of all events in which birds were detected near solar towers.

Event	Date	Time	Burn?	Comment	Cameras
1	5/15/2014	13:00:14	Y	Small bird passes close to receiver, flashes while passing through flux (no smoke), then flies out of scene to north.	WDR, *TS*
2	5/16/2014	8:03:15	Y	Small bird flying on irregular course, flashes while passing through flux (no smoke), then flies out of scene to southeast.	WDR, *TS*
3	5/16/2014	8:19:25	Y	Presumed small bird circles close near receiver then hovers, while flashing (no smoke), and then disappears behind top of receiver.	WDR
4	5/17/2014	3:33:39	N	Small flock (approx. 5-15) of birds flying above tower at night.	**TS**
5	5/17/2014	7:15:07	N	Small yellow bird quickly passes receiver with no obvious flashing or smoke.	WRD, **TS**
6	5/19/2014	0:19:58	N	Large bird (likely egret) flying a few meters above the cameras.	**TS**
7	9/2/2014	23:08:39	N	Small flock (approx. 20) of birds flying past tower at night.	**TS**
8	9/3/2014	8:52:33	N	Small yellow bird dives quickly towards tower with no obvious flashing or smoke.	WDR, **TS**
9	9/3/2014	8:55:56	Y	Small bird circling above tower that then descends into flux , flashes, and flies out of scene to west.	WDR, **TS**
10	9/3/2014	8:58:22	N	Presumed large bird only detected with thermal surveillance camera flying high above tower.	**TS**
11	9/3/2014	9:00:00	N	Medium-sized bird (likely falcon) flies toward tower above flux, circles once, then flies out of scene to south.	WDR, **TS**

12	9/3/2014	9:02:38	N	Medium-sized to large-sized bird makes quick pass above tower flying from north to south.	WDR, **TS**
13	9/3/2014	9:04:19	Y	Small bird (possibly hummingbird) flies in toward receiver, begins flashing as it hovers erratically, then descends with smoke to north.	WDR, **TS**
14	9/3/2014	9:12:20	Y	Small bird passes above receiver to south and flashes slightly while passing through flux to west.	WDR, **TS**
15	9/3/2014	9:28:05	Y	Small bird (possibly hummingbird) hovers through flux west of tower, flashing brightly and occasionally smoking as it flies out of scene to north.	WDR
16	9/3/2014	11:22:37	N	Large bird (likely hawk) circling above flux to west of tower.	WDR, **TS**
17	9/4/2014	7:21:45	N	Small bird makes a looping flight near the non-fluxing receiver approximately level with the upper deck.	WDR, **TS**
18	9/4/2014	19:36:20	N	Medium-sized bird (likely nighthawk) flies a few meters above the cameras.	**TS**
19	9/5/2014	5:22:58	N	Small flock (5-10) of birds flying past and above tower at night.	**TS**
20	9/5/2014	7:00:50	N	Large bird (likely raven) passes just above top of the non-fluxing receiver.	WDR, **TS**
21	9/5/2014	9:43:07	N	Large bird flying west of tower and out of range of all cameras except thermal surveillance.	**TS**
22	9/5/2014	10:41:19	Y	Small bird flying northward through flux to west of tower, flashing slightly.	WDR, **TS**, SGT
23	9/5/2014	10:59:09	N	Large birds (n=3) circle above flux to northwest then leave scene to north.	WDR, **TS**

24	9/5/2014	11:31:03	N	Medium-sized birds (n=2; likely pigeons) approach and land on upper deck of fluxing tower, roost for about 14 minutes, depart briefly, then return.	WDR, **TS**
25	9/5/2014	11:46:45	N	Small bird emerges from south side of tower below upper deck and circles around to west.	WDR, **TS**
26	9/5/2014	11:52:47	N	Medium-sized birds (n=2) circle northwest of tower above the flux.	WDR, TS
27	9/6/2014	10:10:50	N	Large birds (n=3; likely ravens) passing and/or circling in airspace north and west of tower.	WDR, **TS**
28	9/6/2014	10:11:51	N	Large bird (likely gull) bird passes close to fluxing receiver (shadow seen) on south side of tower.	WDR, **TS**
29	9/9/2014	12:22:58	N	Medium- to large-sized bird circling above flux and tower to north and west, barely detectable except with thermal surveillance camera.	WDR, **TS**
30	9/9/2014	12:24:16	N	Small- to medium-sized bird passes somewhere below level of upper deck.	WDR, **TS**
31	9/9/2014	13:29:20	N	Large bird (likely raven) flying northward above flux to west of tower.	WDR, **TS**
32	9/10/2014	8:57:20	Y	Small bird rapidly approached receiver while flashing, then disappeared with smoke over top.	WDR, **TS**
33	9/10/2014	10:10:47	N	Medium-sized bird circling above flux to west and north of tower.	WDR, **TS**
34	9/10/2014	10:33:31	Y	Small bird flies in close to receiver, flashing and smoking near receiver, before flying out of scene to north.	WDR, **TS**
35	9/10/2014	12:34:55	N	Large flock (aprox. 50-75) of birds flies above the flux and tower heading east-southeast.	WDR, **TS**

36	9/11/2014	10:12:10	Y	Medium-sized bird appears in flux from north of tower, flashing and smoking as it flies out of scene to west.	WDR, **TS**
37	9/11/2014	16:51:22	N	Large birds (n=2; Ravens) circling above flux to west of tower.	WDR, **TS**
