# Supplementary material for: Evaluating the Effectiveness of Wildlife Detection and Observation Technologies at a Solar Power Tower Facility
Source: PLoS One. 2016 Jul 27;11(7):e0158115. doi: 10.1371/journal.pone.0158115 (PMC4963080; doi:10.1371/journal.pone.0158115)
Supplement: S3 Table — Also showing stations with greatest and second greatest number of insects captured in parentheses. Samples collected in the top portions of the two-part malaise trap are labeled as “T” for top, whereas samples collected from bottom portions are labeled “B” for bottom. Multiple stations that shared an equal number of insects collected are listed together. (PDF) [file pone.0158115.s007.pdf]

S3 Table. Total number of insects collected during each daytime and nighttime sampling period in September 2014.

	Total number of arthropods		Station and greatest count of insects in parentheses		Station and 2nd greatest count of insects in parentheses	
Date (2014)	Daytime sampling	Nighttime sampling	Daytime sampling	Nighttime sampling	Daytime sampling	Nighttime sampling
15-May	34		5B (9)			
16-May	84	46	5B (15)	1 (10)	9T (11)/10T (11)	5B (8)
17-May	153	52	6 (19)	3 (8)	2B (18) / 7B (18)	10T (7)
18-May	41	52	7T (6)	7T (9)	5B (5)	9T (6) / 10T (6)
19-May	49	36	5B (8)	7B (5)		
20-May	27	42	5B (4)	10T (14)	2B (3) / 10T (3)	7B (4) / 11 (4)
21-May	39	14	9B (7)	7T (2) / 10T (2)	5B (6)	
22-May		22		2B (3) / 4 (3)		
Sum of counts	427	264				
