# Supplementary material for: Evaluating the Effectiveness of Wildlife Detection and Observation Technologies at a Solar Power Tower Facility
Source: PLoS One. 2016 Jul 27;11(7):e0158115. doi: 10.1371/journal.pone.0158115 (PMC4963080; doi:10.1371/journal.pone.0158115)
Supplement: S4 Table — Also showing stations with greatest and second greatest number of insects captured in parentheses. Samples collected in the top portions of the two-part malaise trap are labeled as “T” for top, whereas samples collected from bottom portions are labeled “B” for bottom. Multiple stations that shared an equal number of insects collected are listed together. (PDF) [file pone.0158115.s008.pdf]

S4 Table. Total number of insects collected during each daytime and nighttime sampling period in September 2014.

	Total number of arthropods		Station and greatest count of insects in parentheses		Station and 2nd greatest count of insects in parentheses	
Date (2014)	Daytime sampling	Nighttime sampling	Daytime sampling	Nighttime sampling	Daytime sampling	Nighttime sampling
3-Sep	99	14	5B (19)	8 (8)	6 (14)	
4-Sep	69	44	10T (21)	7T (8)	5B (8)	9T (6)
5-Sep	42	42	9B (8)	10T (12)	5B (6)	7T (6)
6-Sep	55	35	3 (9) /10T (9)	7T (6)	9T (8)	
7-Sep	90	35	5B (19)	7T (9)	7B (12)	2T (6)
8-Sep	*	84	*	5B (27)	*	9T (9)
9-Sep	179	107	5B (66)	9B (14)	9B (17)	10T (12)
10-Sep	130	61	10B (20)	5B (11) /10T (11)	7T (10)	10B (7)
11-Sep	102	43	10T (19)	10T (11)	6 (16)	5B (9)
Sum of counts	766	465				

* Onset of 100-year rainstorm during the day that prevented insect collection
